# Empathy in Facial Mimicry of Fear and Disgust: Simultaneous EMG-fMRI Recordings During Observation of Static and Dynamic Facial Expressions

**DOI:** 10.3389/fpsyg.2019.00701

**Published:** 2019-03-27

**Authors:** Krystyna Rymarczyk, Łukasz Żurawski, Kamila Jankowiak-Siuda, Iwona Szatkowska

**Affiliations:** ^1^Department of Experimental Psychology, Institute of Cognitive and Behavioural Neuroscience, SWPS University of Social Sciences and Humanities, Warsaw, Poland; ^2^Laboratory of Psychophysiology, Department of Neurophysiology, Nencki Institute of Experimental Biology, Polish Academy of Sciences (PAS), Warsaw, Poland

**Keywords:** facial mimicry, EMG, fMRI, mirror neuron system, emotional expressions, dynamic, disgust, fear

## Abstract

Real-life faces are dynamic by nature, particularly when expressing emotion. Increasing evidence suggests that the perception of dynamic displays enhances facial mimicry and induces activation in widespread brain structures considered to be part of the mirror neuron system, a neuronal network linked to empathy. The present study is the first to investigate the relations among facial muscle responses, brain activity, and empathy traits while participants observed static and dynamic (videos) facial expressions of fear and disgust. During display presentation, blood-oxygen level-dependent (BOLD) signal as well as muscle reactions of the corrugator supercilii and levator labii were recorded simultaneously from 46 healthy individuals (21 females). It was shown that both fear and disgust faces caused activity in the corrugator supercilii muscle, while perception of disgust produced facial activity additionally in the levator labii muscle, supporting a specific pattern of facial mimicry for these emotions. Moreover, individuals with higher, compared to individuals with lower, empathy traits showed greater activity in the corrugator supercilii and levator labii muscles; however, these responses were not differentiable between static and dynamic mode. Conversely, neuroimaging data revealed motion and emotional-related brain structures in response to dynamic rather than static stimuli among high empathy individuals. In line with this, there was a correlation between electromyography (EMG) responses and brain activity suggesting that the Mirror Neuron System, the anterior insula and the amygdala might constitute the neural correlates of automatic facial mimicry for fear and disgust. These results revealed that the dynamic property of (emotional) stimuli facilitates the emotional-related processing of facial expressions, especially among whose with high trait empathy.

## Introduction

### Empathy and Facial Mimicry

In the last decade, researchers have focused on empathy as an essential component of human social interaction. The term ‘empathy’ – derived from Greek *empatheia* – ‘passion’ – is a multifaceted construct that is thought to involve both cognitive (i.e., understanding of another’s beliefs and feelings) and affective (i.e., ability to share another’s feelings) components (Jankowiak-Siuda et al., 2011; Betti and Aglioti, 2016). It is believed that people empathize with others by simulating their mental states or feelings. According to the Perception-Action Model (PAM) of empathy, simulative processes discovered and defined in the domain of actions “result from the fact that the subject’s representations of the emotional state are automatically activated when the subject pays attention to the emotional state of the object” (Preston and de Waal, 2002, p. 1; de Waal and Preston, 2017). Paying attention to the other’s emotional state, in turn, leads to the related autonomic and somatic responses (Preston, 2007). Consistent with this model, a positive association between emotional empathy and somatic response was observed for both skin conductance (Levenson and Ruef, 1992; Blair, 1999; Hooker et al., 2008) and cardiac activation (Krebs, 1975; Hastings et al., 2000). This might indicate that more empathic persons react with stronger affective sharing. Recent studies suggest also that empathic traits relate to variation in facial mimicry (FM) (Sonnby-Borgström, 2002; Sonnby-Borgström et al., 2003; Dimberg et al., 2011; Balconi and Canavesio, 2013a, 2014; Rymarczyk et al., 2016b).

Facial mimicry is spontaneous unconscious mirroring of others’ emotional facial expressions, which leads to congruent facial muscles activity (Dimberg, 1982). This phenomenon usually is measured by electromyography (EMG; e.g., Dimberg, 1982; Larsen et al., 2003). Evidence for FM has been most consistently reported when viewing happy (Dimberg and Petterson, 2000; Weyers et al., 2006; Rymarczyk et al., 2011) and angry (Dimberg et al., 2002; Sato et al., 2008) facial expressions. Interestingly, angry facial expressions induce greater activity than happy faces in the corrugator supercilii (CS, muscle involved in frowning), whereas happy facial expressions induce greater activity in the zygomaticus major (ZM, the muscle involved in smiling) and decreased CS activity. In addition, few EMG studies support also the phenomenon of FM for other emotions, i.e., fear with increased activity of CS (e.g., van der Schalk et al., 2011a) or frontalis muscle (e.g., Rymarczyk et al., 2016b) and for disgust with increased activity of CS (e.g., Lundquist and Dimberg, 1995) or levator labii (LL) (e.g., Vrana, 1993). Furthermore, the magnitude of FM has been shown to depend on many factors (for a review see Seibt et al., 2015), including empathic traits (Sonnby-Borgström, 2002; Sonnby-Borgström et al., 2003; Dimberg et al., 2011; Balconi and Canavesio, 2013a; Balconi et al., 2014; Rymarczyk et al., 2016b). For example, Dimberg et al. (2011) have found that more empathic individuals showed greater CS contraction to angry faces and greater ZM contraction to happy faces, as compared to less empathic individuals. Similar patterns were observed in response to fearful facial expressions, where in more empathic individuals exhibited larger CS reactions (Balconi and Canavesio, 2016). Recently, Rymarczyk et al. (2016b) found that emotional empathy moderates activity in other muscles, for instance levator labii in response to disgust and lateral frontalis in response to fearful facial expressions. Results of these studies suggest that more empathic individuals are more sensitive to the emotions expressed by others at the level of facial mimicry. It has been suggested that FM has important consequences for social behavior (Kret et al., 2015) because it facilitates understanding of emotion by inducing an appropriate empathic response (Adolphs, 2002; Preston and de Waal, 2002; Decety and Jackson, 2004).

### Emotional Facial Expression, Mirror Neuron System and Limbic Structures

On the neuronal level, the PAM assumes that observing the actions of another individuals stimulates the same action in the observers by activating the brain structures that are involved in executing the same behavior (Preston, 2007). It has been suggested that the Mirror Neuron System (MNS) represents the neural basis of the PAM (Gallese et al., 1996; Rizzolatti and Sinigaglia, 2010). Indeed, the first evidence of mirror neurons (localized in monkeys in the ventral sector of the F5 area) came from experiments where monkeys performed a goal-directed action (e.g., holding, grasping or manipulating objects) or when they observed another individual (monkeys or human) execute the same action (Gallese et al., 1996; Rizzolatti and Craighero, 2004; Gallese et al., 2009). Similarly, studies in humans have shown that the MNS is activated during imagination or imitation of simple or complex hand movements (Ruby and Decety, 2001; Iacoboni et al., 2005; Iacoboni and Dapretto, 2006). Furthermore, neuroimaging studies have shown that pure observation and imitation of emotional facial expressions engaged the MNS, particularly regions of the inferior frontal gyrus (IFG) and the inferior parietal lobule (IPL) (Rizzolatti et al., 2001; Carr et al., 2003; Rizzolatti and Craighero, 2004; Iacoboni and Dapretto, 2006), which are considered core regions of the MNS in human.

Apart from core regions of the MNS, the insula and the amygdala, limbic system’s structures, are proposed to be involved in processing of emotional facial expressions (Iacoboni et al., 2005). For example the amygdala activation was shown for fear expressions (Carr et al., 2003; Ohrmann et al., 2007; van der Zwaag et al., 2012), while the anterior insula (AI) for disgust expressions (Jabbi and Keysers, 2008; Seubert et al., 2010). Recently, the insula and dorsal part of anterior cingulate cortex together with a set of limbic and subcortical structures (including the amygdala), constitute the brain’s salience network (Seeley et al., 2007). The salience network is thought to mediate the detection and integration of behaviorally relevant stimuli (Menon and Uddin, 2010) including stimuli that elicit fear (Liberzon et al., 2003; Zheng et al., 2017).

Taking into account involvement of the MNS in social mirroring and phenomenon of facial mimicry, the interactions between the MNS and limbic system is postulated (Iacoboni et al., 2005). It is proposed that during observation and imitation of emotional expressions, the core regions of the MNS (i.e., IFG and IPL) activate the insula, which further activate other structure of limbic system, i.e., amygdala (Jabbi and Keysers, 2008). However, it should be emphasize that the specific function of the amygdala in affective resonance is still under debate (Adolphs, 2010). For example, van der Gaag et al. (2007) found bilateral anterior insula activation during perception of happy, disgusted and fearful facial expressions compared to non-emotional facial expressions, however, they did not find any amygdala activation. The amount of studies revealed that amygdala is activated rather during conscious imitation than pure observation of emotional facial expressions (Lee et al., 2006; van der Gaag et al., 2007; Montgomery and Haxby, 2008). Moreover, it was shown that extent of amygdala activation could be predicted by extent of movement during imitation of facial expressions (Lee et al., 2006). Some authors proposed that amygdala activation during imitation, but not observation, of emotional facial expressions might reflect increased autonomic activity or feedback from facial muscles to the amygdala (Pohl et al., 2013).

To sum up, there is general agreement that exists among researchers that the insula is involved in affective resonance. Furthermore, the insula and the amygdala were proposed to be a part of an emotional perception-action matching system (Iacoboni and Dapretto, 2006; Keysers and Gazzola, 2006) and therefore to “extend” the classical MNS during emotion processing (van der Gaag et al., 2007; Likowski et al., 2012; Pohl et al., 2013). It is believed that the mirror mechanism might be responsible for motor simulation of facial expressions (core MNS, i.e., IFG and IPL) (Carr et al., 2003; Wicker et al., 2003; Grosbras and Paus, 2006; Iacoboni, 2009), and for affective imitation (extended MNS, i.e., insula) (van der Gaag et al., 2007; Jabbi and Keysers, 2008). However, the exactly role of the amygdala in these processes is not clear.

### MNS, FM and Empathy

According to the Perception-Action Model, the facial mimicry is an automatic matched motor response, based on a perception-behavior link (Chartrand and Bargh, 1999; Preston and de Waal, 2002). However, other authors proposed that that FM is not only a simple motor reaction, but also a result of a more generic processes of interpreting the expressed emotion (Hess and Fischer, 2013, 2014). Some evidence for this proposition comes from two studies that used simultaneous measures of blood-oxygen level-dependent (BOLD) and facial electromyography (EMG) signals in an MRI scanner (Likowski et al., 2012; Rymarczyk et al., 2018). Likowski et al. (2012) have found that, for emotional facial expressions of happiness, sadness, and anger, facial EMG correlated with BOLD activity localized to parts of the core MNS (i.e., IFG), as well as areas responsible for processing of emotion (i.e., AI). Similar results were obtained in a separate study that additionally utilized videos of happiness and anger facial expressions were also used (Rymarczyk et al., 2018). In that study, Rymarczyk et al. (2018) showed that activation in core MNS and MNS-related structures were more frequently observed when dynamic emotional expressions were presented as compared to static emotional expressions presentations. The authors concluded that dynamic emotional facial expressions might be a clearer signal to induce motor simulation processes in the core the MNS as well as the affective resonance processes in limbic structure, i.e., insula. It is worth noting that dynamic stimuli, as compared to static, selectively activated structures related to motion and biological motion perception (Arsalidou et al., 2011; Foley et al., 2012; Furl et al., 2015), as well as MNS brain structures (Sato et al., 2004; Kessler et al., 2011; Sato et al., 2015). Results of aforementioned EMG-fMRI studies suggest that the core MNS and MNS-related limbic structures (e.g., insula) may constitute neuronal correlates of FM. Furthermore, it appears that FM phenomenon contains a motor and an emotional component, each represented by a specific neural network of active brain structures that correlated with facial muscle responses during perception of emotions. Responsible for the motor component are structures thought to be the one constituting the core MNS (e.g., inferior frontal gyrus), involved in observation and execution of motor actions. The insula, MNS-related limbic structure, is involved in emotional-related processes. It should be noted that this assumption is restricted to FM for happiness, sadness, and anger emotion, based on the results of EMG-fMRI studies.

Furthermore, several studies have linked empathic traits to neural activity in the MNS indicating that individuals who have higher activity in the MNS also score higher on emotional aspects of empathy (Kaplan and Iacoboni, 2006; Jabbi et al., 2007; Pfeifer et al., 2008). For example, Jabbi et al. (2007) found positive correlation between the bilateral anterior insula and the frontal operculum activation when subjects observed video clips displaying pleased or disgusted facial expressions. To sum up, there is some evidence that the MNS is underpinning of empathy and that subsystems of MNS is supporting motor and affective simulation. However, till now there is no empirical evidence for link between the MNS, empathy and simulation processes.

### Aims of the Study

In our study simultaneous recording of EMG and BOLD signal during perception of facial stimuli were used. We selected natural, static and dynamic facial expressions (neutral, fear, and disgust) from the Amsterdam Dynamic Facial Expression Set (ADFES) (van der Schalk et al., 2011b), based on studies showing that dynamic stimuli are a truer reflection of real-life situations (Krumhuber et al., 2013; Sato et al., 2015; Rymarczyk et al., 2016a). Empathy levels were assessed with the Questionnaire Measure of Emotional Empathy (QMEE), wherein empathy is defined as a “vicarious emotional response to the perceived emotional experiences of others” (Mehrabian and Epstein, 1972, p. 1). According to the reasoning outlined above, our EMG-fMRI investigation had two main goals.

Firstly, we wanted to explore whether the neuronal bases for FM, established for socially related stimuli, i.e., anger and happiness, would be the same for more biologically relevant ones, i.e., fear and disgust. We predicted, that similarly to anger and happiness, the core MNS (i.e., IFG and IPL) and MNS-related limbic structures (i.e., insula, amygdala) would be involved in perception of emotional facial expression. Since, that there is evidence that perception of dynamic emotional stimuli elicits greater brain activity as compared to static stimuli (Arsalidou et al., 2011; Kessler et al., 2011; Foley et al., 2012; Furl et al., 2015), we expected the stronger activation in all structures of MNS subsystems for dynamic compared to static emotional facial expression.

Secondly, based on the evidence that empathy traits modulate facial mimicry for fear (Balconi and Canavesio, 2016) and disgust (Balconi and Canavesio, 2016; Rymarczyk et al., 2016b), as well as based on the assumption that MNS is the underpinning of empathy processes, we wanted to test whether there are a relations between facial mimicry, empathy and the mirror neuron system. We predicted that highly empathic people would be characterized with greater activation of extended MNS sites, i.e., insula and amygdala, and that these activations would be correlated with stronger facial reactions. Next, according to neuroimaging evidence that the dynamic compared to static emotional stimuli are stronger signal for social communication (Bernstein and Yovel, 2015; Wegrzyn et al., 2015), we explored whether the relations between facial mimicry, empathy and subsystems of MNS could be also be dependant on the modality of the stimuli.

## Materials and Methods

### Subjects

Forty-six healthy individuals (25 males, 21 females, mean ± standard deviation age = 23.8 ± 2.5 years) participated in this study. The subjects had normal or corrected to normal eyesight and none reported neurological diseases. This study was carried out in accordance with the recommendations of Ethics Committee of Faculty of Psychology at the University of Social Sciences and Humanities with written informed consent from all subjects. All subjects gave written informed consent in accordance with the Declaration of Helsinki. The protocol was approved by the Ethics Committee at the SWPS University of Social Sciences and Humanities. An informed consent form was signed by each participant after the experimental procedures had been clearly explained. After the scanning session, subjects were informed of the aims of the study.

### Empathy

Empathy scores were measured with Questionnaire Measure of Emotional Empathy (QMEE), wherein empathy is defined as *a* “vicarious emotional response to the perceived emotional experiences of others” (Mehrabian and Epstein, 1972, p. 1). The QMEE contains 33-items to be completed using a 9-point ratings from -4 (=very strong disagreement) to +4 (=very strong agreement) and was selected given that the questionnaire has a Polish adaptation (Rembowski, 1989) and has been shown to be a useful measure in FM research (Sonnby-Borgström, 2002; Dimberg et al., 2011). For analysis purposes subjects were split into High Empathy (HE) and Low Empathy (LE) groups based on the median score on the QMEE questionnaire.

### Facial Stimuli and Apparatus

**FIGURE 1:**
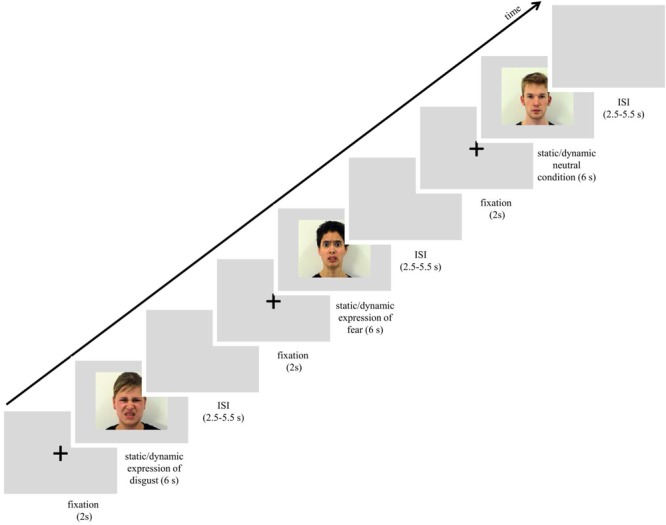
Scheme of procedure used in the study. Images used in the figure were obtained and published with permission of the copyright holder of the Amsterdam Dynamic Facial Expression Set (van der Schalk et al., 2011b).

Facial expressions of disgust and fear were taken from The Amsterdam Dynamic Facial Expression Set (van der Schalk et al., 2011b). Additionally, neutral conditions of the same human actors were used, showing no visible action units specific to emotional facial expression. Stimuli (F02, F04, F05, M02, M08, and M12) consisted of forward-facing facial expressions presented as static and dynamic displays. Stimuli in the static condition consisted of a single frame from the dynamic video clip, corresponding to its condition. For static fear and disgust, the selected frame represented the peak moment of facial expression. In the case of neutral dynamic expressions, motion was still apparent because actors were either closing their eyes or slightly changing the position of their head. Stimuli were 576 pixels in height and 720 pixels in width. All expressions were presented on a gray background. For an overview of procedure and stimuli see Figure 1.

### EMG Acquisition

Electromyography data were acquired using an MRI-compatible Brain Products’ BrainCap consisting of 2 bipolar and one reference electrode. The electrodes with a diameter of 2 mm were filled with electrode paste and positioned in pairs over the CS and LL on the left side of the face (Cacioppo et al., 1986; Fridlund and Cacioppo, 1986). A reference electrode, 6 mm in diameter, was filled with electrode paste and attached to the forehead. Before the electrodes were attached, the skin was cleaned with alcohol. This procedure was repeated until electrode impedance was reduced to 5 kΩ or less. The digitized EMG signals were recorded using a BrainAmp MR plus ExG amplifier and BrainVision Recorder. The signal was low-pass filtered at 250 Hz during acquisition. Finally, data were digitized using a sampling rate of 5 kHz, and stored on a computer running MS Windows 7 for offline analysis.

### Image Acquisition

The MRI data were acquired on a Siemens Trio 3 T MR-scanner equipped with a 12-channel phased array head coil. Functional MRI images were collected using a T2^∗^-weighted EPI gradient-echo pulse sequence with the following parameters: TR = 2,000 ms, TE = 25 ms; 90° flip angle, FOV = 250 mm, matrix = 64 × 64, voxel size = 3.5 mm × 3.5 mm × 3.5 mm, interleaved even acquisition, slice thickness = 3.5 mm, 39 slices.

### Procedure

Each volunteer was introduced to the experimental procedure and signed a consent form. To conceal the true purpose, facial electromyography recordings, participants were told that sweat gland activity was being recorded while watching the faces of actors selected for commercials by an external marketing company. Following the attachment of the electrodes of the FaceEMGCap-MR, participants were reminded to carefully observe the actors presented on the screen and were positioned in the scanner. The subjects were verbally encouraged to feel comfortable and behave naturally.

The scanning session started with a reminder of the subject’s task. In the session subjects were presented with 72 trials that lasted approximately 15 min. Each trial started with a white fixation cross, 80 pixels in diameter, which was visible for 2 s in the center of the screen. Next, one of the stimuli with a facial expression (disgusts, fear or neutral, each presented as static image or dynamic video clip) was presented for 6 s. The expression was followed by a blank gray screen presented for 2.75–5.25 s (see Figure 1). All stimuli were presented in the center of the screen. In summary, each stimulus was repeated once, for a total of 6 presentations within a type of expression (e.g., 6 dynamic presentations of happiness). The stimulus appeared in an event-related manner, pseudo-randomized trial by trail with constraints in rand no facial expression from the same actor, and no more than 2 actors of the same sex or the same emotion were presented consecutively. In total, 6 randomized event-related sessions with introduced constraints were balanced between subjects. The procedure was controlled using Presentation^®^ software running on a computer with Microsoft Windows operating system and was displayed on a 32-inch NNL LCD MRI-compatible monitor with a mirroring system (1920 pixels × 1080 pixels resolution; 32 bit color rate; 60 Hz refresh rate) from a viewing distance of approximately 140 cm.

### Data Analysis

#### EMG Analysis

Pre-processing was carried out using BrainVision Analyzer 2 (version 2.1.0.327). First, EPI gradient-echo pulse artifacts were removed using the average artifact subtraction AAS method (Allen et al., 2000) implemented in the BrainVision Analyzer. This method is based on the sliding average calculation, and consists of 11 consecutive functional volumes marked in the data logs. Synchronization hardware and MR trigger markers allowed for the use of the AAS method for successfully removing MR-related artifacts from the data. Next, standard EMG processing was carried out, which included a signal transformation with 30 Hz high-pass filter. The EMG data were subsequently rectified and integrated over 125 ms and resampled to 10 Hz. Artifacts related to EMG were detected using two methods. First, when single muscle activity was above 8 μV at baseline (i.e., visibility of the fixation cross) (Weyers et al., 2006; Likowski et al., 2008, 2011), the trial was classified as an artifact and excluded from further analysis. All remaining trials were blind-coded and visually checked for artifacts. In the next step, trials were baseline corrected such that the EMG response was measured as the difference of averaged signal activity between the stimuli duration (6 s) and baseline period (2 s). Finally, the signal was averaged for each condition, for each participant. These averaged values were subsequently imported into SPSS 21 for statistical analysis.

Differences in EMG responses were examined using a three-way mixed-model ANOVA with expression (disgust, fear, and neutral) and stimulus mode (dynamic and static) as within-subjects factors and empathy group [low empathy (LE), high empathy (HE)] as the between-subjects factor^1^. Separate ANOVAs were calculated for responses from each muscle, and reported with a Bonferroni correction and with Greenhouse-Geisser correction, when the sphericity assumption was violated. In order to confirm that EMG activity changed from baseline and that FM occurred, EMG data for each significant effect were tested for a difference from zero (baseline) using one-sample, two-tailed *t*-tests.

#### fMRI Processing and Analysis

Image processing and analysis was carried out using SPM12 software (6470) run in MATLAB 2013b (The Mathworks Inc, 2013). Standard pre-processing steps were applied to functional images, i.e., motion-correction and co-registration to the mean functional image. The independent SPM segmentation module was used to divide structural images into different tissue classes [gray matter, white matter, and non-brain (cerebrospinal fluid, skull)]. Next, based on previously segmented structural images, a study-specific template was created and affine registered to MNI space using the DARTEL algorithm. In particular, the functional images were warped to MNI space based on DARTEL priors, resliced to 2 mm × 2 mm × 2 mm isotropic voxels and later smoothed with an 8 mm × 8 mm × 8 mm full-width at half maximum Gaussian kernel. Single subject design matrices were constructed with six experimental conditions, corresponding to dynamic and static trials for each of the three expression conditions (disgust, fear, and neutral). These conditions were modeled with a standard hemodynamic response function, as well as, other covariates including head movements and parameters that excluded other fMRI artifacts produced by Artifact Detection Toolbox (ART). Later, the same sets of contrasts of interest (listed under “Results” section, i.e., fMRI data) were calculated for each subject and used in group level analysis (i.e., one-sample *t*-test) for statistical Regions of Interest (ROIs) analysis. The analysis was performed using the MarsBar toolbox (Brett et al., 2002) for the individual ROIs. ROIs consisted of anatomical masks derived from the WFU Pickatlas (Wake Forest University, 2014), and SPM Anatomy Toolbox (Eickhoff, 2016). The STS was defined as an overlapping set of peaks with a radius of 8 mm based on activation peaks reported in literature (Van Overwalle, 2009). Each ROI was extracted as the mean value from the mask. Statistics of brain activity in each contrast were reported with Bonferroni correction.

#### Correlation Analysis

To understand mutual relationship between brain activity and the facial muscle activity and reveal which ROIs are directly related to FM, bootstrapped (BCa, samples = 1000) Pearson correlation coefficients were calculated between contrasts of brain activity (disgust dynamic, disgust static, fear dynamic, and fear static) and corresponding mimicry.

Each ROI was represented by a single value, which was the mean of all the voxels in that anatomical mask in each hemisphere. Muscle activity was defined as baseline corrected EMG trials of the same muscle and type. The correlations were performed in pairs of variables of muscle and EMG activity, e.g., CS response to static disgust faces with fMRI response in the left insula to static disgust faces.

## Results

### Empathy Scores

The QMEE scores of the two groups were significantly different [*t*_(44)_ = 9.583, *p* < 0.001; *M*_HE_ = 69.4, *SE*_HE_ = 3.7; *M*_LE_ = 14,64, *SE*_LE_ = 4.3]. The HE group included 13 males (*M* = 61.38, *SE* = 4.86) and 11 females (*M* = 78.91, *SE* = 4.42) and the LE group consisted of 12 males (*M* = 12.83, *SE* = 6.35) and 10 females (*M* = 16.8, *SE* = 6.18).

### EMG Measures

#### M. Corrugator Supercilii

ANOVA^2^ showed a significant main effect of expression [*F*_(2,72)_ = 26.527, *p* < 0.001, η^2^ = 0.424], indicating that activity of the CS for disgust (*M* = 0.217, *SE* = 0.025) was similar to fear [*M* = 0.216, *SE* = 0.020; *t*_(36)_ = 0.036, *p* > 0.999] and higher for both fear and disgust as compared to neutral expressions [*M* = 0.028, *SE* = 0.018; disgust vs. neutral: *t*_(36)_ = 5.559, *p* < 0.001; fear vs. neutral: *t*_(36)_ = 6.714, *p* < 0.001]. Between-subject effect of empathy were also significant [*F*_(1,36)_ = 24.813, *p* < 0.001, η^2^ = 0.408] with the activity of CS generally higher for HE (*M* = 0.215, *SE* = 0.016) than LE (*M* = 0.092, *SE* = 0.019) group.

**FIGURE 2:**
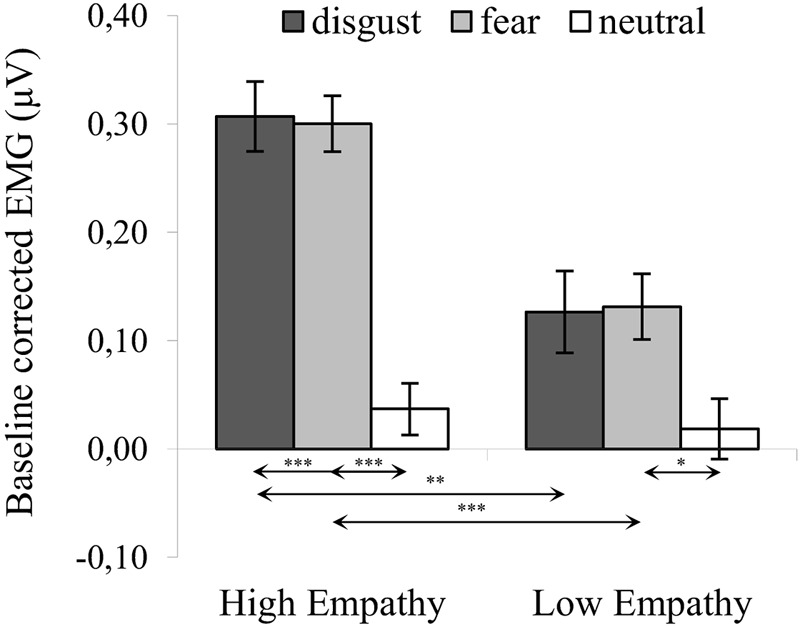
Mean (±SE) EMG activity changes and corresponding statistics for corrugator supercilii during presentation conditions. Separate asterisks indicate significant differences between conditions (simple effects) in EMG responses: ^∗^*p* < 0.05, ^∗∗^*p* < 0.01, ^∗∗∗^*p* < 0.001. SE, standard error.

The significant interaction of expression × empathy group [*F*_(2,72)_ = 4.583, *p* = 0.013, η^2^ = 0.113] revealed that activity of the CS in the HE group for disgust (*M* = 0.307, *SE* = 0.032) was similar to fear [*M* = 0.300, *SE* = 0.026; *t*_(36)_ = 0.194, *p* > 0.999] and higher for both emotions compared to neutral expressions [*M* = 0.037, *SE* = 0.024; disgust vs. neutral: *t*_(36)_ = 6.136, *p* < 0.001; fear vs. neutral: *t*_(35)_ = 7.306, *p* < 0.001]. In the LE, in contrast, higher CS activity was observed for fear (*M* = 0.131, *SE* = 0.030) compared to neutral expressions [*M* = 0.019, *SE* = 0.028; *t*_(36)_ = 2.690, *p* = 0.034] and no other pair differences were observable [*M_LE_*_disgust_ = 0.126, *SE_LE_*_disgust_ = 0.038; LE: disgust vs. neutral: *t*_(36)_ = 2.118, *p* = 0.128; LE: disgust vs. fear: *t*_(36)_ = 0.119, *p* > 0.999]. Higher CS activity was observed in the HE group as compared to the LE group for disgust [*t*_(36)_ = 3.620, *p* = 0.001] and fearful faces [*t*_(36)_ = 4.225, *p* < 0.001]. No group differences observed for neutral expressions [*t*_(36)_ = 0.486, *p* = 0.621] (see Figure 2).

There was no significant main effect of modality [*F*_(1,36)_ = 0.169, *p* = 0.683, η^2^ = 0.005] and the following interactions did not reach significance: modality × empathy [*F*_(1,36)_ = 0.044, *p* = 0.834, η^2^ = 0.001], expression × modality [*F*_(2,72)_ = 0.013, *p* = 0.987, η^2^ = 0.000] and expression × modality × empathy [*F*_(2,72)_ = 0.039, *p* = 0.962, η^2^ = 0.001].

One-sample *t*-tests in HE and LE groups revealed a significant increase in CS activity for all disgust and fear conditions (see Table 1). There was no significant difference in CS activity from baseline in response to neutral expressions.

**Table 1:** Descriptive statistics for corrugator supercilii activity.

		*M*	*SE*	*t*	*p*
Disgust	HE	0.31	0.04	8.77	0.000
	LE	0.14	0.03	5.16	0.000
Fear	HE	0.29	0.03	9.06	0.000
	LE	0.13	0.02	7.78	0.000
Neutral	HE	0.04	0.02	1.48	0.154
	LE	0.02	0.02	0.86	0.405

*HE, high empathy group; LE, low empathy group; M, mean; SE, standard error;t, value of one sample t-test if value in column M differs from baseline; p, p-value, indicating if one sample t-test is significant, i.e., if value in column M significantly differs from baseline.*

#### M. Levator Labii

ANOVA^3^ showed a significant main effect of expression [*F*_(2,76)_ = 33.989, *p* < 0.001, η^2^ = 0.486], indicating that activity of the LL was higher for disgust (*M* = 0.170, *SE* = 0.022) as compared to both fear [*M* = -0.073, *SE* = 0.031; *t*_(36)_ = 6.914, *p* < 0.001] and neutral expressions [*M* = -0.073, *SE* = 0.025; *t*_(36)_ = 8.483, *p* < 0.001]. There was no difference in LL activity between fear and neutral conditions [*t*_(36)_ = 0.105, *p* > 0.999]. The between-subject effect of empathy was significant [*F*_(1,36)_ = 6.579, *p* = 0.015, η^2^ = 0.155], such that activity of LL was higher for HE (*M* = 0.052, *SE* = 0.023) compared to LE (*M* = -0.038, *SE* = 0.026) groups.

**FIGURE 3:**
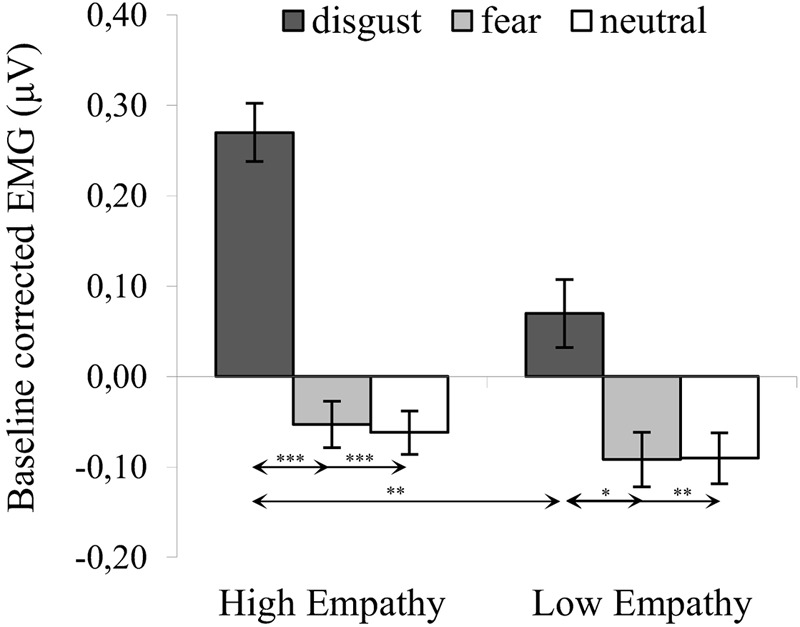
Mean (±SE) EMG activity changes and corresponding statistics for levator labii during presentation conditions. Separate asterisks indicate significant differences between conditions (simple effects) in EMG responses: ^∗^*p* < 0.05, ^∗∗^*p* < 0.01, ^∗∗∗^*p* < 0.001. SE, standard error.

The significant interaction of expression × empathy group [*F*_(2,72)_ = 3.980, *p* = 0.023, η^2^ = 0.100] revealed that, for HE group, activity of the LL was higher for disgust (*M* = 0.270, *SE* = 0.028) compared to both fear [*M* = -0.053, *SE* = 0.040; *t*_(36)_ = 7.022, *p* < 0.001] and neutral expressions [*M* = -0.062, *SE* = 0.033; *t*_(36)_ = 8.973, *p* < 0.001]. Similarly, in the LE group, higher LL activity was observed for disgust (*M* = 0.070, *SE* = 0.033) compared to fear [*M* = -0.092, *SE* = 0.030; *t*_(36)_ = 2.981, *p* = 0.014] and neutral expressions [*M* = -0.090, *SE* = 0.039; *t*_(36)_ = 3.636, *p* = 0.003] (see Figure 3). Within groups, there was no difference in LL between fear and neutral expressions [HE: *t*_(36)_ = 0.184, *p* > 0.999; LE: *t*_(36)_ = 0.034, *p* > 0.999].

The main effect of modality was not significant [*F*_(1,36)_ = 1.315, *p* = 0.259, η^2^ = 0.035] and the following interactions did not reach significance: modality × empathy [*F*_(1,36)_ = 0.000, *p* = 0.995, η^2^ = 0.000], expression × modality [*F*_(2,72)_ = 0.458, *p* = 0.634, η^2^ = 0.013] and expression × modality × empathy [*F*_(2,72)_ = 0.238, *p* = 0.789, η^2^ = 0.007].

One-sample *t*-tests in HE and LE groups revealed higher LL activity for all disgust conditions as compared to baseline (see Table 2). There was no differences in LL activity from baseline in response to fear and neutral expressions.

**Table 2:** Descriptive statistics for levator labii activity.

		*M*	*SE*	*t*	*p*
Disgust	HE	0.27	0.03	8.53	0.000
	LE	0.09	0.02	3.52	0.003
Fear	HE	–0.06	0.04	–1.44	0.164
	LE	–0.08	0.04	–2.13	0.048
Neutral	HE	–0.06	0.03	–2.05	0.053
	LE	–0.08	0.04	–1.76	0.097

*HE, high empathy group; LE, low empathy group; M, mean; SE, standard error; t, value of one sample t-test if value in column M differs from baseline; p, p-value, indicating if one sample t-test is significant, i.e., if value in column M significantly differs from baseline.*

### fMRI Data

Regions of interest analyses were carried out for the contrasts that compare brain activation while viewing dynamic versus static facial expressions, resulting in eleven contrasts of interest: disgust dynamic > disgust static, fear dynamic > fear static, neutral dynamic > neutral static, emotion dynamic > emotion static (emotion dynamic – pooled dynamic disgust, and fear conditions; emotion static – similar pooling), all dynamic > all static (all dynamic – pooled dynamic disgust, fear and neutral conditions; all static – similar pooling), disgust dynamic > neutral dynamic, disgust static > neutral static, fear dynamic > neutral dynamic, fear static > neutral static, emotion dynamic > neutral dynamic, emotion static > neutral static. The aforementioned contrasts were calculated in order to investigate two types of questions. The contrast emotion/disgust/fear/all dynamic/static > neutral dynamic/static addresses neural correlates of FM of emotional/disgust/fear/all expressions. The other contrasts (i.e., emotion/disgust/fear/all dynamic > emotion/disgust/fear/all static) relate to the difference in processing between dynamic and static stimuli. Due to no group differences between HE and LE subjects, we report only fMRI ROI results for all subjects (for corresponding whole brain analysis see Supplementary Tables).

Regions of interest analyses identified activation in the right hemisphere for the disgust dynamic > disgust static contrast (see Table 3).

**Table 3:** Summary statistics for activation in each ROI across all participants for disgust dynamic > disgust static contrast.

	Left Hemisphere			Right Hemisphere		
Region of Interest	*M*	*t*	*p*	*M*	*t*	*p*
V5/MT+	0.445	11.098	0.000***	0.732	9.419	0.000***
Primary Motor Cortex	–0.043	–0.976	0.833	–0.038	–0.927	0.821
Premotor Cortex	–0.003	–0.079	0.531	0.020	0.615	0.271
Inferior Parietal Lobule	0.000	0.006	0.498	0.025	0.708	0.241
Superior Temporal Sulcus	0.255	7.797	0.000***	0.301	8.313	0.000***
BA44	0.034	0.996	0.162	0.022	0.786	0.218
BA45	0.051	1.371	0.089	0.084	2.969	0.002+
Amygdala	0.083	2.435	0.009	0.085	2.774	0.004
Anterior Cingulate Cortex	–0.044	–1.590	0.941	–0.048	–1.745	0.956
Anterior Insula	0.065	2.733	0.004	0.044	2.133	0.019
Caudate Head	0.014	0.399	0.346	0.020	0.565	0.288
Putamen	0.020	0.765	0.224	0.006	0.244	0.404
Globus Pallidus	0.031	1.517	0.068	0.029	1.354	0.091

*Asterisks indicate significant, Bonferroni corrected, activations of each ROI: ^+^p < 0.1, ^∗∗^p < 0.01, ^∗∗∗^p < 0.001.*

Bilateral activation was observed in the V5/MT+ and STS for the fear dynamic > fear static contrast. Moreover in the right hemisphere BA45, amygdala and AI were activated (see Table 4).

**Table 4:** Summary statistics for activation in each ROI across all participants for fear dynamic > fear static contrast.

	Left Hemisphere			Right Hemisphere		
Region of Interest	*M*	*t*	*p*	*M*	*t*	*p*
V5/MT+	0.401	8.114	0.000***	0.748	12.919	0.000***
Primary Motor Cortex	–0.079	–2.084	0.979	–0.051	–1.405	0.917
Premotor Cortex	–0.049	–1.610	0.943	–0.012	–0.406	0.657
Inferior Parietal Lobule	–0.033	–0.869	0.805	0.025	0.624	0.268
Superior Temporal Sulcus	0.245	7.639	0.000***	0.362	11.236	0.000***
BA44	0.055	1.404	0.084	0.001	0.032	0.487
BA45	0.113	2.438	0.009	0.121	3.196	0.001*
Amygdala	0.088	2.458	0.009	0.102	2.882	0.003+
Anterior Cingulate Cortex	–0.003	–0.106	0.542	0.006	0.210	0.417
Anterior Insula	0.073	2.536	0.007	0.075	2.958	0.002+
Caudate Head	0.009	0.266	0.396	0.035	0.986	0.165
Putamen	0.020	0.719	0.238	0.010	0.411	0.341
Globus Pallidus	0.008	0.351	0.363	0.027	1.407	0.083

*Asterisks indicate significant, Bonferroni corrected, activations of each ROI: ^+^p < 0.1, ^∗^p < 0.05, ^∗∗^p < 0.01, ^∗∗∗^p < 0.001.*

For the neutral dynamic > neutral static contrast, only V5/MT+ and STS were activated bilaterally (see Table 5).

**Table 5:** Summary statistics for activation in each ROI across all participants for neutral dynamic > neutral static contrast.

	Left Hemisphere			Right Hemisphere		
Region of Interest	*M*	*t*	*p*	*M*	*t*	*p*
V5/MT+	0.153	3.212	0.001*	0.341	5.245	0.000***
Primary Motor Cortex	–0.011	–0.255	0.600	–0.012	–0.267	0.605
Premotor Cortex	0.022	0.700	0.244	0.021	0.628	0.267
Inferior Parietal Lobule	0.062	1.679	0.050	0.089	2.156	0.018
Superior Temporal Sulcus	0.103	3.126	0.002*	0.168	4.055	0.000**
BA44	–0.026	–0.575	0.716	0.016	0.416	0.340
BA45	0.036	0.671	0.253	0.079	2.015	0.025
Amygdala	–0.021	–0.620	0.731	0.016	0.614	0.271
Anterior Cingulate Cortex	–0.007	–0.228	0.589	0.001	0.041	0.484
Anterior Insula	0.001	0.043	0.483	0.018	0.585	0.281
Caudate Head	0.061	1.488	0.072	0.094	2.566	0.007
Putamen	0.010	0.339	0.368	0.014	0.544	0.295
Globus Pallidus	0.030	1.474	0.074	0.012	0.568	0.286

*Asterisks indicate significant, Bonferroni corrected, activations of each ROI: ^+^p < 0.1, ^∗^p < 0.05, ^∗∗^p < 0.01, ^∗∗∗^p < 0.001*.

Regions of interest analysis for the emotion dynamic > emotion static contrast, revealed bilateral activations in V5/MT+, STS, AI and amygdala. Other structures activated by this contrast were right BA45 and left AI (see Table 6).

**Table 6:** Summary statistics for activation in each ROI across all participants for emotion dynamic > emotion static contrast.

	Left Hemisphere			Right Hemisphere		
Region of Interest	*M*	*t*	*p*	*M*	*t*	*p*
V5/MT+	0.846	11.680	0.000***	1.481	12.925	0.000***
Primary Motor Cortex	–0.121	–1.954	0.972	–0.089	–1.505	0.930
Premotor Cortex	–0.052	–1.029	0.846	0.008	0.176	0.431
Inferior Parietal Lobule	–0.032	–0.570	0.714	0.051	0.896	0.187
Superior Temporal Sulcus	0.500	9.325	0.000***	0.663	12.771	0.000***
BA44	0.090	1.581	0.060	0.023	0.427	0.336
BA45	0.164	2.531	0.007	0.205	3.623	0.000*
Amygdala	0.170	3.074	0.002+	0.187	3.773	0.000**
Anterior Cingulate Cortex	–0.047	–1.090	0.859	–0.042	–0.912	0.817
Anterior Insula	0.138	3.654	0.000*	0.119	3.381	0.001*
Caudate Head	0.023	0.415	0.340	0.056	0.918	0.182
Putamen	0.040	0.897	0.187	0.016	0.405	0.344
Globus Pallidus	0.039	1.275	0.104	0.056	1.808	0.039

*Asterisks indicate significant, Bonferroni corrected, activations of each ROI: ^+^p < 0.1, ^∗^p < 0.05, ^∗∗^p < 0.01, ^∗∗∗^p < 0.001*.

The all dynamic > all static contrast, indicated bilateral activations in V5/MT+, STS, amygdala and AI. The right BA45 was also activated (see Table 7).

**Table 7:** Summary statistics for activation in each ROI across all participants for all dynamic > all static expressions contrast.

	Left Hemisphere			Right Hemisphere		
Region of Interest	*M*	*t*	*p*	*M*	*t*	*p*
V5/MT+	0.999	11.740	0.000***	1.822	11.546	0.000***
Primary Motor Cortex	–0.132	–1.994	0.974	–0.102	–1.415	0.918
Premotor Cortex	–0.029	–0.552	0.708	0.029	0.553	0.291
Inferior Parietal Lobule	0.029	0.459	0.324	0.140	2.247	0.015
Superior Temporal Sulcus	0.603	10.616	0.000***	0.830	12.137	0.000***
BA44	0.064	0.929	0.179	0.039	0.587	0.280
BA45	0.200	2.631	0.006	0.283	4.016	0.000**
Amygdala	0.149	2.909	0.003+	0.203	4.526	0.000***
Anterior Cingulate Cortex	–0.054	–1.126	0.867	–0.040	–0.864	0.804
Anterior Insula	0.139	3.111	0.002+	0.136	2.900	0.003+
Caudate Head	0.084	1.268	0.106	0.150	2.273	0.014
Putamen	0.050	0.986	0.165	0.030	0.639	0.263
Globus Pallidus	0.068	2.135	0.019	0.068	2.289	0.013

*Asterisks indicate significant, Bonferroni corrected, activations of each ROI: ^+^p < 0.1, ^∗^p < 0.05, ^∗∗^p < 0.01, ^∗∗∗^p < 0.001*.

Regions of interest analysis for the disgust dynamic > neutral dynamic contrast, revealed bilateral activations in V5/MT+, STS and BA45. Other structures revealed by this contrast were left BA44 and left AI (see Table 8).

**Table 8:** Summary statistics for activation in each ROI across all participants for disgust dynamic > neutral dynamic contrast.

	Left Hemisphere			Right Hemisphere		
Region of Interest	*M*	*t*	*p*	*M*	*t*	*p*
V5/MT+	0.379	7.154	0.000***	0.440	6.654	0.000***
Primary Motor Cortex	0.060	1.021	0.156	0.072	1.268	0.106
Premotor Cortex	0.057	1.283	0.103	0.074	1.652	0.053
Inferior Parietal Lobule	0.070	1.560	0.063	0.036	0.745	0.230
Superior Temporal Sulcus	0.226	5.144	0.000***	0.180	4.452	0.000***
BA44	0.145	3.149	0.001*	0.084	2.594	0.006
BA45	0.153	3.281	0.001*	0.099	3.157	0.001*
Amygdala	0.114	2.761	0.004	0.096	2.652	0.006
Anterior Cingulate Cortex	–0.018	–0.533	0.702	–0.026	–0.775	0.779
Anterior Insula	0.131	3.905	0.000**	0.055	2.083	0.021
Caudate Head	0.008	0.175	0.431	–0.003	–0.052	0.521
Putamen	0.061	1.975	0.027	0.057	2.210	0.016
Globus Pallidus	0.007	0.327	0.373	0.035	1.481	0.073

*Asterisks indicate significant, Bonferroni corrected, activations of each ROI: ^+^p < 0.1, ^∗^p < 0.05, ^∗∗^p < 0.01, ^∗∗∗^p < 0.001*.

Regions of interest analysis for the disgust static > neutral static contrast, showed activations in left IPL and right BA45(see Table 9).

**Table 9:** Summary statistics for activation in each ROI across all participants for disgust static > neutral static contrast.

	Left Hemisphere			Right Hemisphere		
Region of Interest	*M*	*t*	*p*	*M*	*t*	*p*
V5/MT+	0.087	2.050	0.023	0.048	0.831	0.205
Primary Motor Cortex	0.092	2.133	0.019	0.098	2.065	0.022
Premotor Cortex	0.082	2.317	0.013	0.075	2.044	0.023
Inferior Parietal Lobule	0.132	3.582	0.000*	0.099	2.110	0.020
Superior Temporal Sulcus	0.073	2.300	0.013	0.047	1.385	0.086
BA44	0.084	1.961	0.028	0.078	2.323	0.012
BA45	0.138	2.861	0.003	0.094	3.058	0.002+
Amygdala	0.010	0.266	0.396	0.027	0.811	0.211
Anterior Cingulate Cortex	0.020	0.625	0.268	0.023	0.680	0.250
Anterior Insula	0.067	2.353	0.012	0.028	1.324	0.096
Caudate Head	0.056	1.700	0.048	0.071	1.943	0.029
Putamen	0.050	1.729	0.045	0.064	2.358	0.011
Globus Pallidus	0.005	0.211	0.417	0.018	0.718	0.238

*Asterisks indicate significant, Bonferroni corrected, activations of each ROI: ^+^p < 0.1, ^∗^p < 0.05, ^∗∗^p < 0.01, ^∗∗∗^p < 0.001*.

For the fear dynamic > neutral dynamic contrast, activations were visible bilaterally in V5/MT+, STS, BA45, amygdala and AI. Activation was also noted in the left BA44 and right putamen (see Table 10).

**Table 10:** Summary statistics for activation in each ROI across all participants for fear dynamic > neutral dynamic contrast.

	Left Hemisphere			Right Hemisphere		
Region of Interest	*M*	*t*	*p*	*M*	*t*	*p*
V5/MT+	0.310	5.995	0.000***	0.470	8.129	0.000***
Primary Motor Cortex	0.010	0.250	0.402	0.020	0.486	0.315
Premotor Cortex	0.015	0.467	0.321	0.046	1.501	0.070
Inferior Parietal Lobule	0.033	0.842	0.202	0.016	0.409	0.342
Superior Temporal Sulcus	0.213	5.166	0.000***	0.269	7.614	0.000***
BA44	0.153	3.569	0.000*	0.073	2.084	0.021
BA45	0.184	3.181	0.001*	0.134	3.510	0.001*
Amygdala	0.187	4.699	0.000***	0.165	5.454	0.000***
Anterior Cingulate Cortex	0.014	0.464	0.323	0.010	0.360	0.360
Anterior Insula	0.171	4.755	0.000***	0.098	3.806	0.000**
Caudate Head	0.008	0.203	0.420	0.016	0.436	0.332
Putamen	0.075	2.691	0.005	0.072	3.260	0.001*
Globus Pallidus	0.034	1.457	0.076	0.055	2.538	0.007

*Asterisks indicate significant, Bonferroni corrected, activations of each ROI: ^+^p < 0.1, ^∗^p < 0.05, ^∗∗^p < 0.01, ^∗∗∗^p < 0.001*.

For the fear static > neutral static contrast, activations were observed in the left IPL and left AI (see Table 11).

**Table 11:** Summary statistics for activation in each ROI across all participants for fear static > neutral static contrast.

	Left Hemisphere			Right Hemisphere		
Region of Interest	*M*	*t*	*p*	*M*	*t*	*p*
V5/MT+	0.062	1.509	0.069	0.062	1.438	0.079
Primary Motor Cortex	0.078	1.988	0.026	0.059	1.495	0.071
Premotor Cortex	0.087	2.680	0.005	0.078	2.688	0.005
Inferior Parietal Lobule	0.128	3.402	0.001*	0.079	2.063	0.022
Superior Temporal Sulcus	0.071	2.287	0.013	0.075	2.218	0.016
BA44	0.072	1.544	0.065	0.087	2.663	0.005
BA45	0.107	1.855	0.035	0.092	2.420	0.010
Amygdala	0.078	2.199	0.017	0.079	2.447	0.009
Anterior Cingulate Cortex	0.010	0.347	0.365	0.005	0.144	0.443
Anterior Insula	0.099	2.929	0.003+	0.041	1.573	0.061
Caudate Head	0.060	1.313	0.098	0.075	1.665	0.051
Putamen	0.065	2.030	0.024	0.075	2.481	0.008
Globus Pallidus	0.055	2.102	0.021	0.040	1.577	0.061

*Asterisks indicate significant, Bonferroni corrected, activations of each ROI: ^+^p < 0.1, ^∗^p < 0.05, ^∗∗^p < 0.01, ^∗∗∗^p < 0.001*.

The emotion dynamic > neutral dynamic contrast indicated bilateral activations in V5/MT+, STS, BA45, amygdala and AI. Activation was also observed in the left BA44 and right putamen for this contrast (see Table 12).

**Table 12:** Summary statistics for activation in each ROI across all participants for emotion dynamic > neutral dynamic contrast.

	Left Hemisphere			Right Hemisphere		
Region of Interest	*M*	*t*	*p*	*M*	*t*	*p*
V5/MT+	0.689	7.126	0.000***	0.909	7.935	0.000***
Primary Motor Cortex	0.070	0.748	0.229	0.092	0.996	0.162
Premotor Cortex	0.073	1.030	0.154	0.120	1.731	0.045
Inferior Parietal Lobule	0.104	1.342	0.093	0.051	0.660	0.256
Superior Temporal Sulcus	0.438	5.496	0.000***	0.449	6.412	0.000***
BA44	0.298	3.748	0.000**	0.156	2.612	0.006
BA45	0.337	3.682	0.000**	0.232	3.805	0.000**
Amygdala	0.301	4.085	0.000**	0.261	4.335	0.000**
Anterior Cingulate Cortex	–0.004	–0.068	0.527	–0.016	–0.302	0.618
Anterior Insula	0.302	4.765	0.000***	0.152	3.248	0.001*
Caudate Head	0.016	0.208	0.418	0.013	0.170	0.433
Putamen	0.136	2.523	0.008	0.128	2.978	0.002+
Globus Pallidus	0.040	1.055	0.149	0.090	2.235	0.015

*Asterisks indicate significant, Bonferroni corrected, activations of each ROI: ^+^p < 0.1, ^∗^p < 0.05, ^∗∗^p < 0.01, ^∗∗∗^p < 0.001*.

The emotion static > neutral static contrast was associated with activation in left premotor cortex, left IPL, and right BA45(see Table 13).

**Table 13:** Summary statistics for activation in each ROI across all participants for emotion static > neutral static contrast.

	Left Hemisphere			Right Hemisphere		
Region of Interest	*M*	*t*	*p*	*M*	*t*	*p*
V5/MT+	0.149	2.041	0.024	0.111	1.296	0.101
Primary Motor Cortex	0.170	2.474	0.009	0.156	2.096	0.021
Premotor Cortex	0.169	2.915	0.003+	0.153	2.786	0.004
Inferior Parietal Lobule	0.259	4.121	0.000**	0.178	2.496	0.008
Superior Temporal Sulcus	0.144	2.624	0.006	0.122	2.240	0.015
BA44	0.156	1.898	0.032	0.166	2.733	0.004
BA45	0.245	2.443	0.009	0.185	2.898	0.003+
Amygdala	0.088	1.371	0.089	0.106	1.889	0.033
Anterior Cingulate Cortex	0.030	0.558	0.290	0.028	0.479	0.317
Anterior Insula	0.167	2.935	0.003+	0.069	1.685	0.049
Caudate Head	0.116	1.723	0.046	0.146	2.071	0.022
Putamen	0.115	2.107	0.020	0.139	2.754	0.004
Globus Pallidus	0.061	1.300	0.100	0.058	1.278	0.104

*Asterisks indicate significant, Bonferroni corrected, activations of each ROI: ^+^p < 0.1, ^∗^p < 0.05, ^∗∗^p < 0.01, ^∗∗∗^p < 0.001.*

### Correlation Analysis

#### Muscle-Brain Correlations of Dynamic and Static Disgust Conditions in All Subjects

Correlation analyses in all subjects revealed linear relationships in the disgust dynamic condition between left AI and LL. In the disgust static condition, a positive relationship was present between the LL and activation of the right premotor cortex, and right caudate head. In the left hemisphere, positive relationships were found between the LL and activation in BA44, BA45, and AI (see Table 14).

**Table 14:** Muscles-brain correlations of dynamic and static disgust conditions in all subjects.

	Disgust Dynamic				Disgust Static			
	CS		LL		CS		LL	
Region of Interest	LH	RH	LH	RH	LH	RH	LH	RH
V5/MT+	–0.094	–0.219	0.033	0.045	0.162	0.003	–0.057	0.141
Primary Motor Cortex	0.169	0.204	0.115	–0.024	0.161	0.153	0.181	0.177
Premotor Cortex	0.156	0.139	0.193	0.160	0.236	0.223	0.317*	0.276+
Primary Somatosensory Cortex	0.165	0.069	0.153	–0.035	0.213	0.156	0.182	0.161
Inferior Parietal Lobule	0.141	–0.031	0.181	0.067	0.358*	0.216	0.193	0.009
Superior Temporal Sulcus	0.086	–0.067	0.194	0.248	0.337*	0.176	0.107	–0.040
BA44	–0.014	–0.070	0.052	–0.176	0.025	0.113	0.299+	0.187
BA45	0.062	–0.046	0.031	–0.028	0.147	0.086	0.308+	0.219
Amygdala	0.163	0.214	0.156	0.154	0.246	0.114	0.235	0.142
Anterior Cingulate Cortex	–0.140	–0.141	0.086	0.105	0.279+	0.241	–0.009	–0.011
Anterior Insula	0.259	0.072	0.285+	0.228	0.317*	0.214	0.306+	0.235
Caudate Head	0.020	0.108	0.189	0.210	0.437**	0.476**	0.253	0.276+
Putamen	0.063	0.090	0.191	0.132	0.150	0.215	0.156	0.173
Globus Pallidus	–0.076	0.138	0.059	0.220	0.339*	0.274+	0.240	0.259

*Post-number asterisks indicate significant Pearson correlations of muscle-ROI pairs: ^+^p < 0.1, ^∗^p < 0.05, ^∗∗^p < 0.01. CS, corrugator supercilii; LL, levator labii; LH, left hemisphere; RH, right hemisphere.*

Positive relationships between CS and brain activity was found in the right hemisphere in the caudate head and globus pallidus as well as in various regions of the left hemisphere (IPL, STS, ACC, AI, caudate head, globuspallidus) (see Table 14).

#### Muscle-Brain Correlations of Dynamic and Static Fear Conditions in All Subjects

Correlation analyses in all subjects revealed a positive relationship between CS in activation in the left BA44, right BA45, and AI for the static fear condition. In the dynamic fear condition, there was a positive relationship between CS and activation in the left globus pallidus (see Table 15).

**Table 15:** Muscles-brain correlations of dynamic and static fear conditions in all subjects.

	Fear Dynamic		Fear Static	
Region of Interest	CS		CS	
	LH	RH	LH	RH
V5/MT+	–0.099	0.042	–0.049	–0.112
Primary Motor Cortex	0.063	0.034	–0.038	–0.003
Premotor Cortex	–0.019	–0.024	0.128	0.126
Primary Somatosensory Cortex	0.050	0.050	–0.038	–0.007
Inferior Parietal Lobule	–0.034	–0.016	0.059	0.120
Superior Temporal Sulcus	0.061	–0.019	0.052	0.029
BA44	–0.049	–0.001	0.281+	0.102
BA45	–0.119	–0.087	0.200	0.200
Amygdala	0.171	0.183	0.213	0.295+
Anterior Cingulate Cortex	–0.028	–0.027	–0.141	–0.087
Anterior Insula	0.007	–0.006	0.201	0.301+
Caudate Head	–0.048	–0.087	0.130	0.152
Putamen	0.060	0.044	0.202	0.246
Globus Pallidus	0.268+	0.005	0.201	0.213

*Post-number asterisks indicate significant Pearson correlations of muscle-ROI pairs: ^+^p < 0.1. CS, corrugator supercilii. LH, left hemisphere; RH, right hemisphere.*

#### Muscle-Brain Correlations of Dynamic and Static Disgust Conditions in High Empathic Subjects

Correlation analyses of dynamic disgust in HE subjects revealed a positive relationship between LL and brain activity in several region of the right (STS, amygdala, AI, caudate head, putamen, globus pallidus) and left hemispheres (amygdala, AI, caudate head, putamen). For static disgust in HE subjects, the relationship between LL and brain activity was significant for the left AI, right caudate head, and bilateral amygdalae (see Table 16).

**Table 16:** Muscles-brain correlations of dynamic and static disgust conditions in high empathic subjects.

	Disgust Dynamic				Disgust Static			
	CS		LL		CS		LL	
Region of Interest	LH	RH	LH	RH	LH	RH	LH	RH
V5/MT+	–0.169	–0.257	–0.011	0.054	–0.071	–0.241	–0.194	0.154
Primary Motor Cortex	0.333	0.379+	0.236	0.093	0.013	0.097	0.286	0.173
Premotor Cortex	0.284	0.232	0.297	0.236	0.181	0.195	0.351	0.319
Primary Somatosensory Cortex	0.407+	0.364+	0.371+	0.202	0.187	0.108	0.269	0.213
Inferior Parietal Lobule	0.131	0.020	0.289	0.296	0.245	0.199	0.209	0.143
Superior Temporal Sulcus	–0.034	0.058	0.150	0.462*	0.215	0.181	0.046	0.145
BA44	0.062	0.013	0.149	–0.147	0.016	0.143	0.314	0.240
BA45	0.033	–0.097	0.077	–0.011	0.036	0.018	0.344	0.302
Amygdala	0.403+	0.391+	0.401+	0.375+	0.395+	0.328	0.426*	0.368+
Anterior Cingulate Cortex	–0.017	–0.099	0.339	0.323	0.327	0.281	0.156	0.186
Anterior Insula	0.318	0.053	0.377+	0.376+	0.332	0.258	0.379+	0.278
Caudate Head	0.114	0.126	0.422+	0.441*	0.560**	0.555**	0.322	0.390+
Putamen	0.255	0.313	0.426*	0.423+	0.368+	0.497*	0.287	0.294
Globus Pallidus	0.115	0.247	0.315	0.428*	0.363+	0.371+	0.193	0.301

*Post-number asterisks indicate significant Pearson correlations of muscle-ROI pairs: ^+^p < 0.1, ^∗^p < 0.05, ^∗∗^p < 0.01. CS, corrugator supercilii; LL, levator labii. LH, left hemisphere; RH, right hemisphere.*

Correlation analyses of dynamic disgust in HE subjects revealed no relationship between CS and brain activations. For the static disgust in HE subjects, the relationship between CS and brain activity was significant in the regions of the right (caudate head, putamen, globus pallidus) and left hemispheres (amygdala, caudate head, putamen, globus pallidus) (see Table 16).

#### Muscle-Brain Correlations of Dynamic and Static Fear Conditions in High Empathic Subjects

Correlation analyses of dynamic fear in HE subjects revealed a positive relationship between CS and brain activity in amygdalae bilaterally and left globus pallidus. For static fear in HE subjects a significant relationship between CS and brain activity was significant for the bilateral amygdalae and putamen and right AI (see Table 17).

**Table 17:** Muscles-brain correlations of dynamic and static fear conditions in high empathic subjects.

	Fear Dynamic		Fear Static	
Region of Interest	CS		CS	
	LH	RH	LH	RH
V5/MT+	–0.227	0.015	–0.122	0.026
Primary Motor Cortex	0.150	0.077	0.010	0.022
Premotor Cortex	–0.040	–0.008	0.177	0.191
Primary Somatosensory Cortex	0.153	0.261	0.018	0.071
Inferior Parietal Lobule	–0.010	0.184	0.082	0.199
Superior Temporal Sulcus	–0.173	0.016	0.018	0.195
BA44	–0.004	0.130	0.276	0.122
BA45	–0.150	–0.039	0.233	0.190
Amygdala	0.368+	0.406+	0.393+	0.411+
Anterior Cingulate Cortex	0.159	0.245	–0.141	–0.113
Anterior Insula	0.045	0.144	0.233	0.369+
Caudate Head	–0.041	–0.095	0.131	0.199
Putamen	0.165	0.217	0.383+	0.426*
Globus Pallidus	0.405+	0.090	0.284	0.332

*Post-number asterisks indicate significant Pearson correlations of muscle-ROI pairs: ^+^p < 0.1. CS, corrugator supercilii. LH, left hemisphere; RH, right hemisphere.*

#### Muscle-Brain Correlations of Dynamic and Static Disgust Conditions in Low Empathic Subjects

In LE subjects, the relationship between LL and brain activity was found only in the disgust static condition, for left BA44, putamen and globus pallidus bilaterally (see Table 18).

**Table 18:** Muscles-brain correlations of dynamic and static disgust conditions in low empathic subjects.

	Disgust Dynamic				Disgust Static			
	CS		LL		CS		LL	
Region of Interest	LH	RH	LH	RH	LH	RH	LH	RH
V5/MT+	–0.111	–0.275	–0.023	0.036	0.523*	0.403	0.139	0.385
Primary Motor Cortex	0.247	0.118	0.206	–0.075	0.389	0.207	0.098	0.166
Premotor Cortex	0.258	0.202	0.344	0.284	0.468+	0.401	0.356	0.316
Primary Somatosensory Cortex	0.226	–0.013	0.231	–0.020	0.381	0.396	0.176	0.340
Inferior Parietal Lobule	0.373	0.207	0.149	–0.120	0.653**	0.669**	0.135	0.052
Superior Temporal Sulcus	0.195	–0.126	0.152	0.175	0.538*	0.508*	0.142	–0.158
BA44	0.069	0.220	0.109	0.123	–0.018	0.221	0.443+	0.321
BA45	0.226	0.098	–0.014	–0.035	0.377	0.277	0.177	0.116
Amygdala	0.378	0.412+	0.330	0.211	0.372	0.135	0.352	0.181
Anterior Cingulate Cortex	0.234	0.354	0.299	0.278	0.610**	0.646**	–0.022	–0.098
Anterior Insula	0.289	0.230	0.247	0.041	0.459+	0.258	0.221	0.324
Caudate Head	0.166	0.383	0.017	0.017	0.454+	0.548*	0.257	0.225
Putamen	0.249	0.338	0.344	0.219	0.139	0.056	0.423+	0.394
Globus Pallidus	–0.478*	0.026	–0.333	–0.156	0.424+	0.379	0.486*	0.610**

*Post-number asterisks indicate significant Pearson correlations of muscle-ROI pairs: ^+^p < 0.1, ^∗^p < 0.05, ^∗∗^p < 0.01. CS, corrugator supercilii; LL, levator labii. LH, left hemisphere; RH, right hemisphere.*

Correlation analyses of dynamic disgust in LE subjects revealed a positive relationship between CS and activity in right amygdala, and negative relationship between this muscle and left globus pallidus. For the static disgust condition, there was a positive relationship between CS and brain activity in the right (IPL, STS, ACC, and caudate head) and in the left hemisphere (V5/MT+, premotor cortex, IPL, STS, ACC, AI, caudate head, globus pallidus) among LE subjects (see Table 18).

#### Muscle-Brain Correlations of Dynamic and Static Fear Conditions in Low Empathic Subjects

In LE subjects, there was a relationship between CS and brain activity only in static fear condition, for left BA44 (see Table 19).

**Table 19:** Muscles-brain correlations of dynamic and static fear conditions in low empathic subjects.

	Fear Dynamic		Fear Static	
Region of Interest	CS		CS	
	LH	RH	LH	RH
V5/MT+	0.049	0.072	0.176	–0.382
Primary Motor Cortex	0.313	0.305	0.191	0.279
Premotor Cortex	0.119	0.178	0.263	0.230
Primary Somatosensory Cortex	0.281	0.207	0.148	0.145
Inferior Parietal Lobule	0.002	0.039	–0.062	0.089
Superior Temporal Sulcus	0.275	0.137	0.117	–0.187
BA44	0.028	0.063	0.461+	0.319
BA45	–0.171	–0.055	0.139	0.196
Amygdala	0.386	0.322	0.398	0.337
Anterior Cingulate Cortex	–0.104	–0.129	0.271	0.389
Anterior Insula	–0.220	–0.235	0.330	0.371
Caudate Head	–0.027	0.126	0.276	0.288
Putamen	0.188	0.186	0.224	0.110
Globus Pallidus	0.025	0.100	0.030	0.086

*Post-number asterisks indicate significant Pearson correlations of muscle-ROI pairs: ^∗^p < 0.05. CS, corrugator supercilii. LH, left hemisphere; RH, right hemisphere.*

## Discussion

In the present study, static and dynamic stimuli were used to investigate facial reactions and brain activation in response to emotional facial expressions. To assess neuronal structures involved in automatic, spontaneous mimicry during perception of fear and disgust facial expressions, we collected simultaneous recordings of the EMG signal and BOLD response during the perception of stimuli. Additionally, to explore whether empathic traits are linked with facial muscle and brain activity, we divided participants into low and high empathy groups (i.e., LE and HE) based on the median score on a validated questionnaire.

The EMG analysis revealed activity in the CS muscle while viewing both fear and disgust facial displays, while perception of disgust induced facial activity specifically in the LL muscle. Moreover, the HE group showed a larger responses in the CS and LL muscles as compared to the LE group, however, these responses were not differentiable between static and dynamic mode of stimuli.

For BOLD data, we used ROI analyses. We found that dynamic emotional expressions elicited higher activation in the bilateral STS, V5/MT+, bilateral amygdalae, and right BA45 as compared to emotional static expression. For the opposite contrast (static > dynamic), as expected, no significant activations emerged.

Using combined EMG-fMRI analysis, we found significant correlations between brain activity and facial muscle reactions for perception of dynamic as well as static emotional stimuli. The correlated brain structures, e.g., amygdala and AI, were more frequent in the HE compared to LE group.

### EMG Response to Fear and Disgust

The main result from EMG recording is that both fear and disgust emotions increased corrugator muscle reactions, whereas levator labii muscle activity was more pronounced in response to disgust than to fearful expressions. Before discussing this result, it should be emphasized that fear and disgust expressions have an opposite biological function, fear is thought to enhance perception to danger and disgust dampens it (Susskind et al., 2008). Accordingly, both emotions are characterized by opposite visible surface features, e.g., faster eye movements or velocity inspiration during perception of fear in comparison to perception of disgust (Susskind et al., 2008). It is suggested that fear and disgust involve opposite psychological mechanisms at the physiological level (Krusemark and Li, 2011). Based on the above-mentioned findings, we anticipated different patterns of facial muscle reaction for the evaluated emotions. Our results concerning the CS contraction for both negative emotions are congruent with earlier studies reporting CS activity during perception of anger (Sato et al., 2008; Dimberg et al., 2011), fear, and disgust emotions (Murata et al., 2016; Rymarczyk et al., 2016b). Moreover, Topolinski and Strack (2015) demonstrated that perception of highly surprising events, compared to lower-level ones, elicited CS activity specifically.

In addition, Neta et al. (2009) suggested that CS activity could reflect the participants’ bias, i.e., tendency to rate surprise as either positive or negative. Thus, it is proposed that CS reactions could be an indicator of a global negative affect (Bradley et al., 2001; Larsen et al., 2003) as well as a tool to measure individual differences in emotion regulatory ability (Lee et al., 2012).

Furthermore, we found increased LL activity for disgust facial expressions, but no evidence of activity for fear presentation. There is some evidence that perception of disgust faces (Vrana, 1993; Lundquist and Dimberg, 1995; Cacioppo et al., 2007; Rymarczyk et al., 2016b), a disgusting picture related to contamination (Yartz and Hawk, 2002) or tasting an unpleasant substance (Chapman et al., 2009) leads to the specific contraction of the LL muscle. Moreover, it was shown that reaction of the LL muscle occurred not only for biological but also moral disgust, i.e., during violation of moral norms (Whitton et al., 2014). Taken together, these results demonstrate the reliability of LL as an indicator of disgust experience (Armony and Vuilleumier, 2013, p. 62).

As far as modality of the stimulus is concerned, we did not observe any differences in the magnitude of facial reactions between static and dynamic stimuli. Similar results were found in our earlier study (Rymarczyk et al., 2016b), wherein reaction of the CS, LL and also lateral frontalis muscles were measured. We showed only a weak impact of dynamic stimuli on the strength of facial reactions for fear expressions. These reactions were apparent only in the lateral frontalis muscle, which was not measured in the present study. It should be noted that most studies have reported higher EMG response during perception of dynamic than static emotional facial expressions (Weyers et al., 2006; Sato et al., 2008; Rymarczyk et al., 2011); however, most of these studies tested the role of dynamic mode on the FM phenomenon for happiness and anger. Together, the role of dynamic stimuli in the FM phenomenon for more biologically embedded emotions needs further research.

### Facial Mimicry and Empathy

Our data also provide some evidence for the relationship between the intensity of FM and trait emotional empathy. We found that HE compared to LE subjects showed stronger activity in CS and LL muscles for fear and disgust. However, the pattern of FM was the same in HE in LE groups. Our results are in agreement with previous EMG studies, wherein researchers have shown that HE subjects show greater mimicry of emotional expressions for happiness and anger (Sonnby-Borgström, 2002; Sonnby-Borgström et al., 2003; Dimberg et al., 2011), as well as, for fear (Balconi and Canavesio, 2016; Rymarczyk et al., 2016b) and disgust (Balconi and Canavesio, 2013b; Rymarczyk et al., 2016b) expressions as compared to LE subjects. Together, these results suggest that FM and emotional empathy are interrelated phenomena (Hatfield et al., 1992; McIntosh, 2006). Moreover, the magnitude of FM may be a strong predictor of empathy. According to PAM (de Waal, 2008), HE people exhibit stronger FM for emotional stimuli because on a neuronal level they engage brain areas related to the representation of their own feelings, for, e.g., the AI (Preston, 2007).

### Neural Network for Fear and Disgust

Neuroimaging data revealed that, observation of dynamic emotional, compared to dynamic neutral stimuli, triggered a distributed brain network that consisted of bilateral STS, V5/MT+, amygdala, AI, and BA45. The left BA44 and right putamen were also activated. In contrast, the perception of static emotional faces as compared to static neutral faces elicitated activity in the left IPL, right BA45, and left AI, and left premotor cortex.

Apart from STS and V5/MT+, greater activity for contrast dynamic vs. static fear was found in the right BA45, right amygdala, and right AI. Dynamic versus static disgust faces induced greater activity in the right BA45. Our findings concerning the bilateral visual area V5/MT+ and STS corroborate previous results confirming the importance of these structures in motion and biological motion perception, respectively (Robins et al., 2009; Arsalidou et al., 2011; Foley et al., 2012; Furl et al., 2015). It has been suggested that, due to their complex features dynamic facial characteristics require enhanced visual analysis in V5/MT+, which might result in wide-spread activation patterns (Vaina et al., 2001).

Previous studies have reported activations in the STS for facial motion due to speech production (Hall et al., 2005), or facial emotional expressions for happiness and anger (Kilts et al., 2003; Rymarczyk et al., 2018), fear (LaBar et al., 2003) and disgust (Trautmann et al., 2009). Moreover, STS activation was reported during detection of movements of natural faces (Schultz and Pilz, 2009), but not computer-generated faces (Sarkheil et al., 2013). According to the neurocognitive model for face processing (Haxby et al., 2000), STS activity could be related to enhanced perceptual and/or cognitive processing for dynamic characteristics of faces (Sato et al., 2004). To summarize our results, together with those of others, support the use of dynamic stimuli to study the neuronal correlates of emotional facial expressions (Fox et al., 2009; Zinchenko et al., 2018).

In our study, we found activity in brain areas typically implicated in simulative process, namely the IFG and IPL (Carr et al., 2003; Jabbi and Keysers, 2008). It has been proposed that understating the behavior of others is based on direct mirroring of somatosensory or motor representations of the observed action in the observer’s brain (Gazzola et al., 2006; van der Gaag et al., 2007; Jabbi and Keysers, 2008). For example, activation of these MNS structures was found during observation and imitation of others actions, i.e., during hand movement (Gallese et al., 1996; Rizzolatti and Craighero, 2004; Molnar-Szakacs et al., 2005; Vogt et al., 2007). Moreover, activity in the IFG was greater during the observation of action-related context as opposed to context-free actions, suggesting this structure plays a role not only in recognition but also in coding the intentions of others (Iacoboni et al., 2005) and contemplating others’ mental states (for meta-analysis see Mar, 2011). Neuroimaging studies have shown involvement of the IFG and IPL during observation of both dynamic and static (Carr et al., 2003) facial stimuli, for example, when comparing dynamic faces to dynamic objects (Fox et al., 2009), dynamic faces to dynamic scrambled faces (Sato et al., 2004; Schultz and Pilz, 2009) and dynamic faces to static faces (Arsalidou et al., 2011; Foley et al., 2012; Rymarczyk et al., 2018). It is interesting that in our study we also found that static compared to neutral images activated IPL and IFG. It is possible that the brain areas involved in the process of motor imagery could be activated also in absence of biological movement, which is typical for emotional but not neutral facial expressions. Accordingly, Kilts et al. (2003) reported that judgment of emotion intensity during perception of both angry and happy static expressions compared to neutral expressions activate motor and premotor cortices. Those authors proposed that during perception of static emotional images “*decoding for emotion content is accomplished by the covert motor simulation of the expression prior to attempts to match the static percept to its dynamic mental representation*” (Kilts et al., 2003, p. 165). To summarize, growing neuroimaging evidence confirms the role of frontal and parietal dorsal streams in the processing of both static (Carr et al., 2003) as well as dynamic emotional stimuli (Sarkheil et al., 2013), also for fear (Schaich Borg et al., 2008) and disgust emotions (Schaich Borg et al., 2008). Since facial emotional expressions are a strong cue in social interactions, it is proposed that natural stimuli (Schultz and Pilz, 2009), especially dynamic ones, may be powerful signals for activating simulation processes within the MNS.

### Relationships Between Facial Muscle Reactions and Neural Activity

In our study, we found that activity in several regions correlated with facial reactions. For fear expressions, CS reactions correlated with activation in the right amygdala, right AI and left BA44 for static displays, and in the left pallidus for dynamic ones. A similar pattern of correlated structures was observed for disgust displays, such that CS reactions correlated with activation in the left AI, left IPL, pallidus, and caudate head bilaterally, for static displays. Moreover, for disgust static displays LL reaction correlated with activation in the left BA44, and left BA45, left AI and bilateral premotor cortex, LL correlations with dynamic displays were primarily observed in the left AI (see Table 14).

In almost all conditions (i.e., during perception of fear and disgust as well as static and dynamic stimuli) facial reactions correlated with activity of brain regions related to motor simulation of facial expressions (i.e., IFG and IPL), as discussed above, as well as in the AI. Similar results were obtained in other studies, wherein simultaneous recording of the EMG signal and BOLD response during perception of stimuli was applied (Likowski et al., 2012; Rymarczyk et al., 2018). For example, Likowski et al. (2012) found that ZM reactions to static happy expressions and CS reactions to static angry faces correlated with activations in the right IFG. Moreover, Rymarczyk et al. (2018) observed such correlations mainly for dynamic stimuli. All together, these studies emphasize the role of the IFG and IPL in intentional imitation of emotional expressions and suggest that these regions, that are sensitive to goal-directed actions, may constitute the neuronal correlates of FM [for a review see, Bastiaansen et al., 2009].

The activation of the AI observed in our study during perception of disgust and fear is in line with the results of other studies (Phan et al., 2002). For example, the AI has been shown to respond during experiences of unpleasant odors (Wicker et al., 2003), tastes (Jabbi et al., 2007), and perception of disgust-inducing pictures (Shapira et al., 2003) as well as disgusted faces (Chen et al., 2009). However, the AI seems to be engaged in processing not only negative but also positive emotions, for, e.g., during smile execution (Hennenlotter et al., 2005). Furthermore, most researchers agree that the AI, which is considered to be structure extending MNS, may underlie a simulation of emotional feeling states (van der Gaag et al., 2007; Jabbi and Keysers, 2008). These assumptions correspond with other findings of simultaneous EMG-fMRI studies that show correlations between insula activity with facial reactions during perception of emotional expressions. For example, Likowski et al. (2012) showed that CS muscle reactions to angry faces were associated with the right insula, while Rymarczyk et al. (2018) found such relationships for happiness expressions with ZM and orbicularis oculi responses. It should be noted that, more recently, the AI is considered to be a key brain region involved in the experience of emotions (Menon and Uddin, 2010), among other processes like judgments of trustworthiness or sexual arousal [for a review see (Bud) Craig, 2009].

Next, in our study we found correlations between activity of the amygdala and facial reactions in the CS muscle during perception of fear stimuli. These results are parallel to other findings of neuroimaging studies that revealed activity of the amygdala during observation (Carr et al., 2003) as well as execution of fear and other negative facial expressions (van der Gaag et al., 2007). A number of studies emphasize the role of the amygdala in social-emotional recognition (Adolphs, 2002; Adolphs and Spezio, 2006), and in particular, in the processing of salient face stimuli during unpredictable circumstances (Adolphs, 2010). Moreover, it has been suggested that the amygdala contributes to relevant stimuli detection (Sander et al., 2003). Therefore, it is possible that, due an increased vigilance in observing the dynamically changing salient features of faces, the processing of dynamic aspects of faces requires amygdala activation.

Furthermore, our EMG-fMRI analysis revealed correlations between activity of the basal ganglia (i.e., globus pallidus and caudate head) and facial reactions for fear and disgust expressions. One interpretation of this result might be that the caudate nucleus and the globus pallidus, which are involved in motor control (Salih et al., 2009), also play a role in motor control during automatic FM. On the other hand, clinical studies (Sprengelmeyer et al., 1996; Calder et al., 2016) and neuroimaging data (Sprengelmeyer et al., 1998) suggest that both the globus pallidus and the caudate nuclei play an important role in processing of disgust expressions. Moreover, the globus pallidus seems to be involved in aversive responses to fear and anxiety (Talalaenko et al., 2008), as well as in affect regulation (Murphy et al., 2003).

### Relationships Between Facial Mimicry, Neural Activity and Empathy

A further innovative feature of our study was to test whether empathy traits modulate the neuronal correlates of FM. As discussed above, the high empathy group as compared to the low empathic one presented a distinct pattern of EMG response that is consistent with a typical FM, i.e., greater CS reactions for fear and disgust and greater LL reactions for disgust. What is important to note here is the FM activity in emotion-related brain structures (e.g., AI, amygdala) was more evident in the HE group. Our finding of the anterior insula activity is partially consistent with few neuroimaging studies where disgust stimuli were used (for a review see Baird et al., 2011). For example it was shown that an observation of film clips of people drinking liquids and displaying disgusted faces evoked activity in a neural circuit consisting of the AI, IFG and cingulate cortex, but only in high empathic persons. It seems that activations related to disgust were more frequently observed for high-arousing stimuli, like pictures of painful situations (Jackson et al., 2006) or facial pain expressions (Botvinick et al., 2005; Saarela et al., 2007). However, in our study, we found no differences in brain activity during perception of fear and disgust facial expressions when comparing low and high empathic subjects. This may be the result from different kind of stimuli used in our and other studies. While most studies used the high-arousing stimuli like the pain-inducing situations, our study applied low arousing stimuli. In other words, the perception of emotional facial expressions, compared to perception of pain-inducing situations may be not sufficient to detect brain differences related to low and high empathic characteristics of subjects.

In relation to correlation between facial reaction for fear and disgust stimuli and activity of the amygdala, our result stay in agreement of the assumption, that the amygdala, next to AI, IFG, and IPL constitute the neuronal structures required for complex empathic processes (Bzdok et al., 2012; Decety et al., 2012; Marsh, 2018). Taken together, it is proposed that activity of the amygdala, together with activity of the insula may constitute the neuronal bases of affective simulation, however, the specificity of role of the amygdala in affective resonance requires further clarification. As noted by Preston and de Waal (2002): “So, if the mirror neurons represent emotional behavior, then the insula may relay information from the premotor mirror neurons to the amygdala” (see Augustine, 1996).

### Summary and Conclusion

Our results from study using simultaneously recorded EMG and BOLD signals during perception of fear and disgust have confirmed that, similarly to anger and happiness (Likowski et al., 2012; Rymarczyk et al., 2018), the MNS may constitute the neuronal bases of FM. In particular, the core MNS structures (i.e., IFG and IPL) are thought to be responsible for motor simulation, while MNS-related limbic regions (e.g., AI) seem to be related to affective resonance. In line with this, it is suggested that FM includes both motor and emotional component; however, their mutual relations required further studies. For example, it is possible that motor imitation leads to emotional contagion or vice versa, among other factors, which play an important role in social interactions.

Our study is the first attempt when the relation between facial mimicry, activity of subsystems of the MNS, and level of emotional empathy was explored. We have found that high empathic people demonstrated the stronger facial reactions and what is worth noting, these reactions were correlated with stronger activation of structures of core MNS and MNS-related limbic structures. In other words, it appears that high empathic people imitate emotions of others more than low empathic ones. Additionally, we have shown that the processes of motor imitation and affective contagion were more evident for dynamic, more natural, than static emotional facial expressions.

As far as modality of the stimuli is concerned, our study confirmed the general agreement that exists among researchers that dynamic facial expressions are a valuable source of information in social communication. The evidence was visible in greater neural network activations during dynamic compared to static facial expressions of fear and disgust. Moreover, it appeared that presentation of stimulus dynamics is an important factor for elicitation of emotion, especially for fear.

### Limitations

As it was noted in the introduction, the increased activity of CS or LL in response to emotional facial expressions are not distinct to single emotions, i.e., neither for fear nor for disgust. Some studies confirmed increased CS activity during perception of various negative emotions (Murata et al., 2016). Accordingly LL increased activity was found not only for in disgust mimicry but also in pain expression, together with increased activity of CS (Prkachin and Solomon, 2008). Therefore, our inference about brain-muscle relationships are limited due to non-specificity of the CS and LL which are indicators of FM for fear and disgust.

Next, there is some evidence that increased activity of other facial muscle, i.e., the lateral frontalis, could be related to fear expression (Van Boxtel, 2010). In our previous work we showed that fear presentations induced activity in this muscle (Rymarczyk et al., 2016b). However, in the current work we did not measure activity of this muscle because the cap intended for EMG measurements in MRI environment was not designed for that purpose.

## Author Contributions

KR, KJ-S, and ŁŻ conceived and designed the experiments. KR and ŁŻ performed the experiments, analyzed the data, and contributed materials. KR, ŁŻ, KJ-S, and IS wrote the manuscript.

## Conflict of Interest Statement

The authors declare that the research was conducted in the absence of any commercial or financial relationships that could be construed as a potential conflict of interest.

## Footnotes

1 Additionally robust 3 way ANOVA based on trimmed means (trim = 0.1) was calculated using WRS2 package (*t3way* function) in R software (version 3.4.0).

2 Robust ANOVA based on trimmed means confirmed the parametric ANOVA results of CS. There were significant main effects of expression (*Q* = 67.741, *p* < 0.001), empathy (*Q* = 31.549, *p* < 0.01) and significant expression × empathy interaction (*Q* = 12.417, *p* < 0.01). No other effects or interactions were significant (*Q*_modality_ = 0.140, *p* > 0.05; *Q*_emotion ×modality_ = 0.144, *p* > 0.05; *Q*_modality × empathy_ = 0.036, *p* > 0.05; Q_emotion × modality × empathy_ = 0.043, *p* > 0.05). Shapiro–Wilk test was used to test normality distribution assumption of levator activity for parametric ANOVA (*W*_HE:disgustdynamic_ = 0.983, *p* > 0.05; *W*_HE:disguststatic_ = 0.940, *p* > 0.05; *W*_HE:feardynamic_ = 0.958, *p* > 0.05; *W*_HE:fearstatic_ = 0.976, *p* > 0.05; *W*_HE:neutraldynamic_ = 0.956, *p* > 0.05; *W*_HE:neutralstatic_ = 0.880, *p* < 0.05; *W*_LE:disgustdynamic_ = 0.955, *p* > 0.05; *W*_LE:disguststatic_ = 0.951, *p* > 0.05; *W*_LE:feardynamic_ = 0.945, *p* > 0.05; *W*_LE:fearstatic_ = 0.992, *p* > 0.05; *W*_LE:neutraldynamic_ = 0.958, *p* > 0.05; *W*_LE:neutralstatic_ = 0.966, *p* > 0.05).

3 Robust ANOVA based on trimmed means confirmed the parametric ANOVA results of levator. There were significant main effects of expression (*Q* = 102.411, *p* < 0.001), empathy (*Q* = 11.668, *p* < 0.01) and significant expression × empathy interaction (*Q* = 12.923, *p* < 0.01). No other effects or interactions were significant (*Q*_modality_ = 2.102, *p* > 0.05; *Q*_emotion ×modality_ = 0.902, *p* > 0.05; *Q*_modality × empathy_ = 0.225, *p* > 0.05;*Q*_emotion × modality × empathy_ = 0.070, *p* > 0.05). Shapiro–Wilk test was used to test normality distribution assumption of levator activity for parametric ANOVA (*W*_HE:disgustdynamic_ = 0.984, *p* > 0.05; *W*_HE:disguststatic_ = 0.941, *p* > 0.05; *W*_HE:feardynamic_ = 0.936, *p* > 0.05; *W*_HE:fearstatic_ = 0.925, *p* > 0.05; *W*_HE:neutraldynamic_ = 0.885, *p* < 0.05; *W*_HE:neutralstatic_ = 0.949, *p* > 0.05; *W*_LE:disgustdynamic_ = 0.937, *p* > 0.05; *W*_LE:disguststatic_ = 0.836, *p* < 0.01; *W*_LE:feardynamic_ = 0.975, *p* > 0.05; *W*_LE:fearstatic_ = 973, *p* > 0.05; *W*_LE:neutraldynamic_ = 0.874, *p* < 0.05; *W*_LE:neutralstatic_ = 0.836, *p* < 0.01).
