# Supplementary material for: Empathy in Facial Mimicry of Fear and Disgust: Simultaneous EMG-fMRI Recordings During Observation of Static and Dynamic Facial Expressions
Source: Front Psychol. 2019 Mar 27;10:701. doi: 10.3389/fpsyg.2019.00701 (PMC6445885; doi:10.3389/fpsyg.2019.00701)

The appendix presents whole brain analysis (results of flexible factorial model) of selected contrasts with corresponding to it visualisation. All contrasts were *p* < 0.05 Family Wise Corrected (FWE), with no additional threshold for a number of voxels activated in the regional cluster (k=1). Abbreviations: L - left hemisphere; R - right hemisphere. Brain labels describing peak activations are reported using SPM Anatomy Toolbox (Eickhoff, 2016).

Supplementary Table 1 Whole brain peak activations for disgust dynamic > disgust static contrast.

| Region | side | k | x | y | z | t |
| --- | --- | --- | --- | --- | --- | --- |
| Middle Temporal Gyrus | R | 2627 | 46 | -66 | 0 | 14,78 |
|  |  |  | 54 | -40 | 8 | 9,88 |
| Inferior Occipital Gyrus | R |  | 34 | -88 | -4 | 5,12 |
| Middle Occipital Gyrus | L | 1506 | -46 | -72 | 0 | 12,16 |
| Middle Temporal Gyrus | L |  | -54 | -52 | 6 | 6,93 |
| Precentral Gyrus | R | 459 | 46 | 2 | 48 | 7,20 |
| IFG (p. Opercularis) | R |  | 48 | 16 | 26 | 4,94 |
| Cerebelum (Crus 2) | L | 42 | -16 | -76 | -36 | 5,85 |
| Fusiform Gyrus | R | 22 | 44 | -50 | -22 | 5,13 |
|  |  |  | 42 | -48 | -18 | 5,02 |


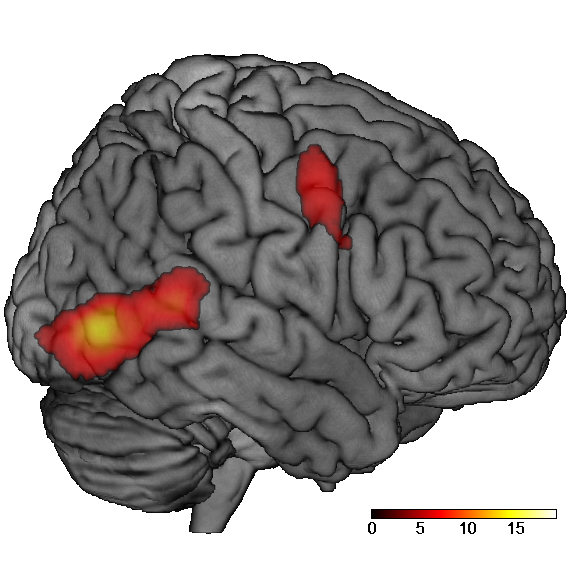


Supplementary Table 2 Whole brain peak activations for fear dynamic > fear static contrast.

| Region | side | k | x | y | z | t |
| --- | --- | --- | --- | --- | --- | --- |
| Middle Temporal Gyrus | R | 2813 | 44 | -62 | 2 | 14,31 |
|  |  |  | 58 | -40 | 8 | 11,31 |
| Middle Occipital Gyrus | L | 1302 | -46 | -70 | 2 | 10,99 |
| Middle Temporal Gyrus | L |  | -54 | -52 | 8 | 7,71 |
|  |  |  | -50 | -44 | 10 | 7,29 |
| IFG (p. Triangularis) | R | 95 | 50 | 26 | -2 | 5,75 |
| Middle Frontal Gyrus | R | 24 | 48 | 4 | 52 | 5,42 |


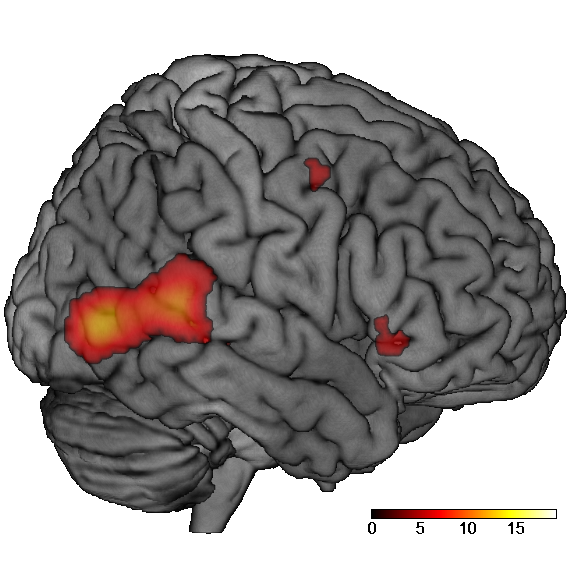


Supplementary Table 3 Whole brain peak activations for neutral dynamic > neutral static contrast.

| Region | side | k | x | y | z | t |
| --- | --- | --- | --- | --- | --- | --- |
|  |  | 765 | 44 | -58 | 2 | 7,88 |
| Superior Temporal Gyrus | R |  | 44 | -38 | 10 | 6,58 |
|  |  |  | 44 | -46 | 8 | 6,28 |
| Superior Temporal Gyrus | R |  | 54 | -40 | 10 | 5,93 |
| Cerebelum (Crus 2) | L | 73 | -18 | -76 | -36 | 5,63 |
| Cerebelum (Crus 1) | L |  | -20 | -68 | -32 | 5,46 |
| Middle Occipital Gyrus | L | 96 | -52 | -74 | 0 | 5,60 |
|  |  |  | -42 | -62 | 6 | 5,52 |
| Thal: Prefrontal | R | 10 | 12 | -6 | 12 | 5,03 |
|  | 0 | 1 | 46 | -16 | -18 | 4,75 |


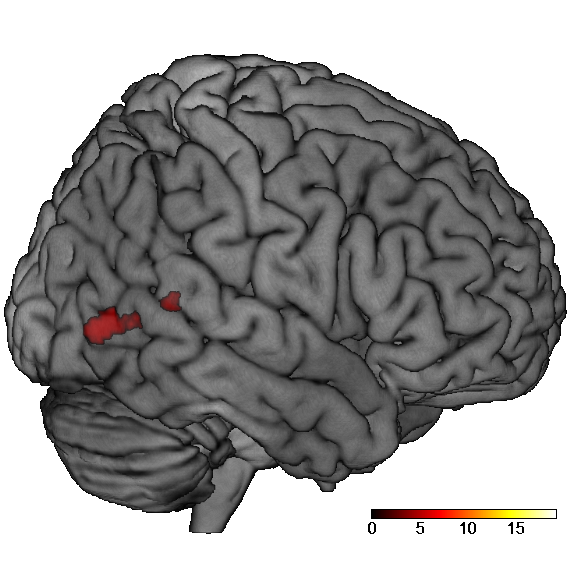


Supplementary Table 4 Whole brain peak activations for emotion dynamic > emotion static contrast.

| Region | side | k | x | y | z | t |
| --- | --- | --- | --- | --- | --- | --- |
| Middle Temporal Gyrus | R | 3371 | 46 | -62 | 2 | 18,28 |
|  |  |  | 56 | -40 | 8 | 13,37 |
| Middle Occipital Gyrus | L | 1793 | -46 | -72 | 0 | 14,55 |
| Middle Temporal Gyrus | L |  | -54 | -52 | 6 | 9,33 |
| Middle Frontal Gyrus | R | 357 | 48 | 4 | 52 | 7,87 |
| Cerebelum (Crus 2) | L | 94 | -18 | -76 | -36 | 6,37 |
| IFG (p. Orbitalis) | R | 79 | 48 | 28 | -4 | 5,87 |
| IFG (p. Triangularis) | R | 73 | 44 | 18 | 24 | 5,76 |
| Fusiform Gyrus | R | 19 | 42 | -48 | -16 | 5,12 |
| Amygdala | R | 13 | 20 | -4 | -16 | 5,08 |
| Medial Temporal Pole | R | 4 | 52 | 12 | -24 | 4,92 |


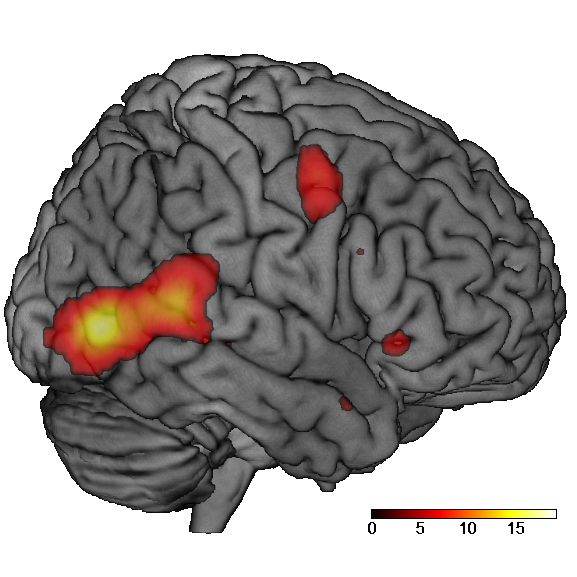


Supplementary Table 5 Whole brain peak activations for all dynamic > all static contrast.

| Region | side | k | x | y | z | t |
| --- | --- | --- | --- | --- | --- | --- |
| Middle Temporal Gyrus | R | 3549 | 44 | -62 | 2 | 18,41 |
|  |  |  | 56 | -40 | 8 | 13,50 |
| Middle Occipital Gyrus | L | 1813 | -46 | -72 | 0 | 14,16 |
| Middle Temporal Gyrus | L |  | -54 | -52 | 6 | 9,01 |
| Cerebelum (Crus 2) | L | 254 | -18 | -76 | -36 | 7,91 |
| Precentral Gyrus | R | 448 | 46 | 2 | 48 | 7,76 |
| IFG (p. Orbitalis) | R | 146 | 48 | 28 | -4 | 5,97 |
| IFG (p. Triangularis) | R | 93 | 44 | 18 | 22 | 5,48 |
| Hippocampus | R | 23 | 20 | -4 | -14 | 5,35 |
| Fusiform Gyrus | R | 13 | 44 | -50 | -20 | 5,16 |
| IFG (p. Opercularis) | R | 3 | 34 | 6 | 32 | 4,91 |
| Medial Temporal Pole | R | 3 | 52 | 12 | -24 | 4,85 |


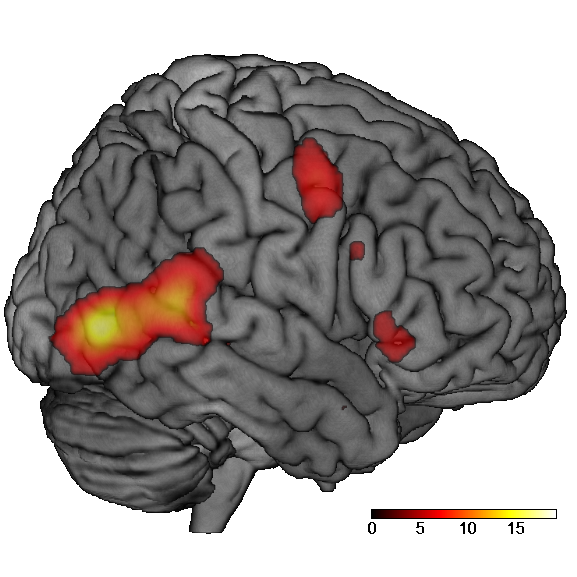


Supplementary Table 6 Whole brain peak activations for disgust dynamic > neutral dynamic contrast.

| Region | side | k | x | y | z | t |
| --- | --- | --- | --- | --- | --- | --- |
| Middle Occipital Gyrus | L | 2406 | -30 | -90 | -4 | 11,40 |
|  |  |  | -46 | -72 | -2 | 10,32 |
| Inferior Temporal Gyrus | L |  | -40 | -46 | -18 | 5,57 |
| Inferior Occipital Gyrus | R | 2594 | 34 | -88 | -4 | 10,40 |
| Middle Temporal Gyrus | R |  | 54 | -66 | -2 | 10,05 |
| Middle Occipital Gyrus | R |  | 36 | -86 | 6 | 9,65 |
| Inferior Occipital Gyrus | R |  | 42 | -72 | -12 | 7,18 |
| Fusiform Gyrus | R |  | 42 | -44 | -20 | 6,73 |
| Precentral Gyrus | R | 264 | 52 | 2 | 44 | 7,58 |
| Middle Temporal Gyrus | R | 230 | 48 | -36 | 4 | 7,27 |
| SupraMarginal Gyrus | R | 132 | 66 | -16 | 28 | 5,73 |
|  |  |  | 64 | -22 | 26 | 5,41 |
|  |  |  | 60 | -24 | 26 | 5,33 |
| Superior Occipital Gyrus | R | 26 | 28 | -84 | 34 | 5,41 |


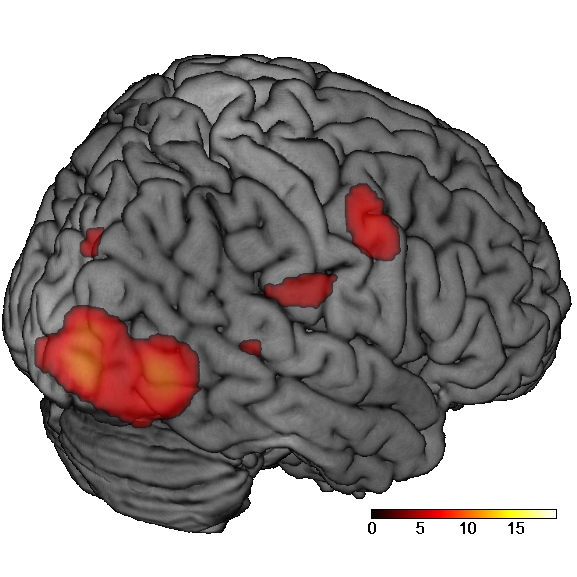


Supplementary Table 7 Whole brain peak activations for disgust static > neutral static contrast.

| Region | side | k | x | y | z | t |
| --- | --- | --- | --- | --- | --- | --- |
| Middle Occipital Gyrus | L | 671 | -30 | -90 | -4 | 7,58 |
| Inferior Occipital Gyrus | L |  | -36 | -88 | -10 | 6,82 |
|  |  |  | -48 | -76 | -12 | 5,60 |
|  |  |  | -46 | -72 | -16 | 5,36 |
|  |  |  | -46 | -68 | -18 | 5,22 |
| Middle Occipital Gyrus | R | 407 | 32 | -88 | 2 | 6,35 |
|  |  |  | 32 | -86 | 6 | 6,27 |
| Inferior Occipital Gyrus | R |  | 36 | -88 | -10 | 6,05 |
| Cerebelum (VI) | L | 144 | -24 | -62 | -22 | 6,28 |
| Thal: Premotor | R | 20 | 26 | -20 | 14 | 5,19 |
| MCC | L | 12 | -6 | 10 | 40 | 5,17 |
| Postcentral Gyrus | R | 6 | 32 | -36 | 70 | 5,00 |
| Cerebelum (VI) | R | 9 | 30 | -56 | -30 | 4,90 |
| Cerebelum (VI) | R | 7 | 20 | -62 | -18 | 4,90 |
| Cerebelum (VI) | L | 7 | -10 | -62 | -20 | 4,87 |
| Cerebelum (VI) | R | 3 | 26 | -54 | -22 | 4,84 |
| Postcentral Gyrus | L | 1 | -24 | -48 | 54 | 4,78 |
| Postcentral Gyrus | R | 1 | 24 | -28 | 58 | 4,74 |


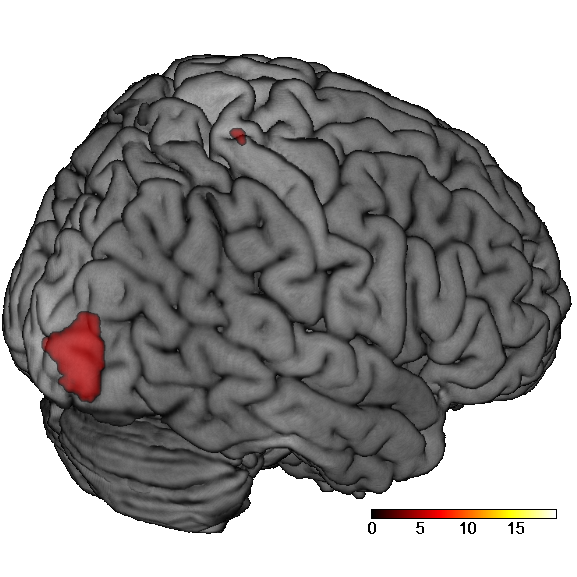


Supplementary Table 8 Whole brain peak activations for fear dynamic > neutral dynamic contrast.

| Region | side | k | x | y | z | t |
| --- | --- | --- | --- | --- | --- | --- |
| Superior Temporal Gyrus | R | 3375 | 52 | -32 | 2 | 9,74 |
| Middle Temporal Gyrus | R |  | 54 | -68 | 0 | 8,95 |
|  |  |  | 58 | -40 | 8 | 8,56 |
|  |  |  | 48 | -68 | 0 | 8,54 |
| Fusiform Gyrus | R |  | 26 | -82 | -8 | 7,71 |
| Superior Temporal Gyrus | R |  | 62 | -32 | 22 | 6,95 |
| Inferior Occipital Gyrus | R |  | 36 | -86 | -6 | 6,72 |
| Middle Occipital Gyrus | R |  | 38 | -88 | 8 | 5,72 |
|  |  |  | 48 | -78 | 10 | 4,88 |
| Middle Occipital Gyrus | L | 1706 | -46 | -72 | 0 | 7,97 |
| Inferior Occipital Gyrus | L |  | -28 | -86 | -10 | 7,73 |
| Middle Temporal Gyrus | L |  | -56 | -56 | 6 | 6,80 |
| SupraMarginal Gyrus | L |  | -52 | -38 | 26 | 5,85 |
| Middle Temporal Gyrus | L |  | -46 | -44 | 8 | 5,10 |
| Fusiform Gyrus | L |  | -38 | -62 | -12 | 5,09 |
| Middle Temporal Gyrus | L |  | -44 | -52 | 6 | 5,06 |
| Fusiform Gyrus | L |  | -40 | -66 | -12 | 5,01 |
| IFG (p. Orbitalis) | R | 315 | 46 | 28 | -4 | 6,96 |
| Temporal Pole | R |  | 50 | 18 | -16 | 5,02 |
| IFG (p. Triangularis) | R |  | 58 | 24 | 4 | 4,95 |
| Precentral Gyrus | R | 130 | 52 | 2 | 48 | 6,70 |
| Fusiform Gyrus | R | 81 | 42 | -42 | -18 | 6,54 |
|  |  |  | 42 | -34 | -18 | 5,35 |
| IFG (p. Orbitalis) | L | 69 | -40 | 22 | -4 | 5,39 |
| Posterior-Medial Frontal | R | 11 | 10 | 8 | 70 | 5,31 |
|  |  |  | 10 | 14 | 68 | 4,98 |
| Amygdala | L | 24 | -20 | -4 | -16 | 5,21 |
| Superior Occipital Gyrus | R | 9 | 18 | -96 | 20 | 5,21 |
| Amygdala | R | 15 | 22 | -4 | -18 | 5,09 |
|  |  |  | 26 | -2 | -20 | 4,89 |
|  | 0 | 8 | -36 | -4 | -16 | 4,99 |
| Posterior-Medial Frontal | R | 1 | 12 | 18 | 66 | 4,77 |
|  | 0 | 2 | 36 | -2 | -18 | 4,76 |
| IFG (p. Opercularis) | L | 1 | -52 | 10 | 6 | 4,76 |


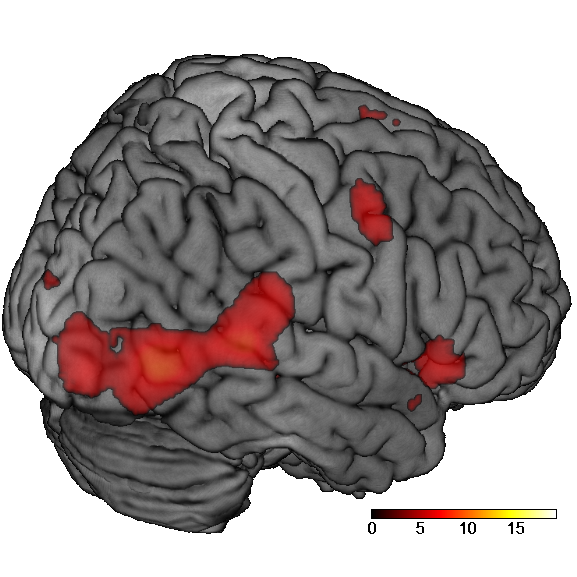


Supplementary Table 9 Whole brain peak activations for fear static > neutral static contrast.

| Region | side | k | x | y | z | t |
| --- | --- | --- | --- | --- | --- | --- |
| Fusiform Gyrus | L | 1775 | -34 | -76 | -18 | 7,76 |
| Cerebelum (VI) | L |  | -30 | -66 | -20 | 7,61 |
| Inferior Occipital Gyrus | L |  | -34 | -86 | -12 | 6,99 |
|  |  |  | -28 | -90 | -10 | 6,84 |
| Inferior Occipital Gyrus | R | 1550 | 34 | -86 | -12 | 7,66 |
|  |  |  | 40 | -76 | -16 | 6,65 |
| Middle Occipital Gyrus | R |  | 30 | -86 | 8 | 6,09 |
| Cerebelum (VI) | R |  | 38 | -50 | -26 | 6,03 |
|  |  |  | 28 | -62 | -18 | 5,41 |
| Fusiform Gyrus | R |  | 44 | -46 | -22 | 5,38 |
| Inferior Temporal Gyrus | R |  | 44 | -42 | -20 | 5,37 |
| Fusiform Gyrus | R |  | 42 | -36 | -20 | 5,35 |
|  |  |  | 28 | -58 | -14 | 5,29 |
| Thalamus | R | 84 | 16 | -10 | 14 | 5,45 |
| Thal: Parietal | R |  | 22 | -22 | 16 | 5,16 |
| SupraMarginal Gyrus | R | 127 | 64 | -28 | 26 | 5,43 |
| Hippocampus | L | 6 | -26 | -26 | -10 | 5,26 |
| Thal: Temporal | R | 14 | 6 | 0 | 4 | 5,07 |
| Thal: Prefrontal | L | 5 | -16 | -6 | 8 | 5,03 |
| Thal: Prefrontal | L | 11 | -8 | -4 | 4 | 4,95 |
|  |  | 4 | -40 | -24 | 26 | 4,88 |
| Area 4p | R | 2 | 40 | -8 | 36 | 4,87 |
| Area 45 | L | 5 | -56 | 18 | -4 | 4,87 |
| IFG (p. Opercularis) | L | 8 | -38 | 8 | 12 | 4,85 |
|  |  | 1 | -32 | 2 | 2 | 4,85 |
| Inferior Temporal Gyrus | L | 1 | -38 | -44 | -16 | 4,85 |
| Posterior-Medial Frontal | R | 2 | 12 | 16 | 66 | 4,79 |
| Insula Lobe | L | 1 | -44 | 14 | 0 | 4,76 |
| Superior Temporal Gyrus | R | 1 | 42 | -36 | 8 | 4,75 |
|  |  | 1 | -30 | 26 | 20 | 4,74 |


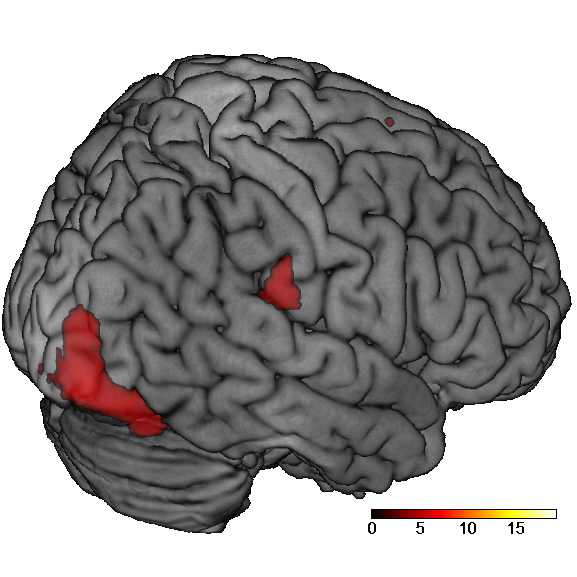


Supplementary Table 10 Whole brain peak activations for emotion dynamic > neutral dynamic contrast.

| Region | side | k | x | y | z | t |
| --- | --- | --- | --- | --- | --- | --- |
| Middle Temporal Gyrus | R | 4126 | 54 | -66 | -2 | 10,65 |
| Inferior Occipital Gyrus | R |  | 34 | -86 | -4 | 9,66 |
| Middle Temporal Gyrus | R |  | 48 | -36 | 4 | 9,46 |
| Middle Occipital Gyrus | R |  | 36 | -86 | 6 | 8,67 |
| Fusiform Gyrus | R |  | 42 | -42 | -18 | 7,40 |
| SupraMarginal Gyrus | R |  | 60 | -30 | 24 | 6,67 |
| Inferior Occipital Gyrus | L | 2605 | -30 | -88 | -8 | 10,27 |
| Middle Occipital Gyrus | L |  | -46 | -72 | -2 | 10,23 |
| Middle Temporal Gyrus | L |  | -54 | -60 | 6 | 7,21 |
| Fusiform Gyrus | L |  | -40 | -66 | -12 | 6,71 |
| Inferior Temporal Gyrus | L |  | -40 | -44 | -16 | 5,44 |
| Precentral Gyrus | R | 252 | 52 | 2 | 46 | 8,03 |
| IFG (p. Orbitalis) | R | 160 | 46 | 26 | -4 | 6,31 |
| IFG (p. Opercularis) | R |  | 50 | 14 | -2 | 4,89 |
| SupraMarginal Gyrus | L | 59 | -50 | -36 | 26 | 5,68 |
| Superior Occipital Gyrus | R | 15 | 26 | -86 | 34 | 5,38 |
| IFG (p. Orbitalis) | L | 25 | -40 | 22 | -4 | 5,15 |
| Posterior-Medial Frontal | R | 3 | 10 | 8 | 70 | 5,13 |
| Amygdala | L | 10 | -20 | -6 | -16 | 5,12 |
| Middle Temporal Gyrus | L | 3 | -46 | -44 | 8 | 4,83 |
| IFG (p. Triangularis) | R | 5 | 58 | 24 | 4 | 4,79 |
| IFG (p. Opercularis) | L | 2 | -52 | 10 | 6 | 4,75 |


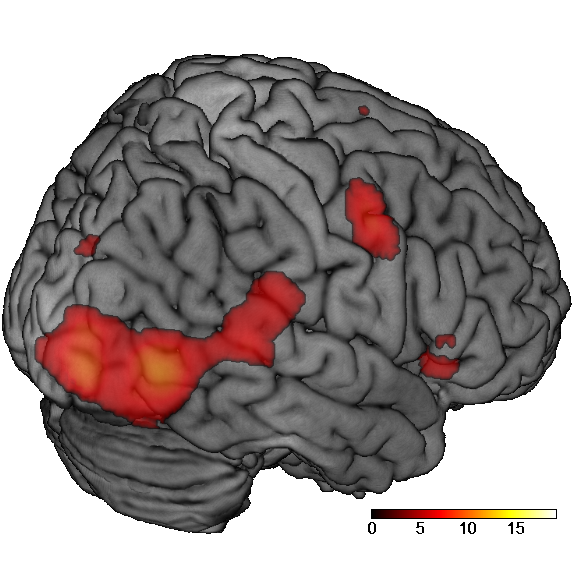


Supplementary Table 11 Whole brain peak activations for emotion static > neutral static contrast.

| Region | side | k | x | y | z | t |
| --- | --- | --- | --- | --- | --- | --- |
| Middle Occipital Gyrus | L | 1889 | -30 | -90 | -4 | 7,98 |
| Inferior Occipital Gyrus | L |  | -36 | -88 | -10 | 7,78 |
| Cerebelum (VI) | L |  | -26 | -64 | -20 | 7,69 |
| Inferior Occipital Gyrus | L |  | -46 | -72 | -16 | 6,40 |
|  |  |  | -42 | -76 | -14 | 6,36 |
|  |  |  | -48 | -76 | -12 | 6,34 |
|  |  |  | -46 | -68 | -18 | 6,29 |
| Cerebelum (VI) | L |  | -10 | -64 | -18 | 4,87 |
| Inferior Occipital Gyrus | R | 1324 | 34 | -88 | -12 | 7,57 |
| Middle Occipital Gyrus | R |  | 32 | -86 | 6 | 6,98 |
| Inferior Occipital Gyrus | R |  | 40 | -76 | -16 | 5,88 |
| Cerebelum (VI) | R |  | 26 | -54 | -22 | 5,62 |
| Inferior Temporal Gyrus | R |  | 44 | -42 | -20 | 5,52 |
| Cerebelum (VI) | R |  | 38 | -50 | -26 | 5,50 |
| Fusiform Gyrus | R |  | 28 | -58 | -14 | 5,34 |
|  |  |  | 44 | -46 | -22 | 5,25 |
| Cerebelum (VI) | R |  | 22 | -62 | -18 | 5,23 |
| Thal: Parietal | R | 106 | 22 | -22 | 16 | 5,80 |
| Thal: Prefrontal | R |  | 18 | -10 | 14 | 4,94 |
| Thalamus | R |  | 14 | -8 | 12 | 4,94 |
| MCC | L | 27 | -8 | 10 | 40 | 5,37 |
| SupraMarginal Gyrus | R | 104 | 62 | -28 | 32 | 5,29 |
|  |  |  | 62 | -30 | 26 | 5,08 |
|  |  |  | 66 | -28 | 24 | 5,03 |
|  |  |  | 68 | -20 | 20 | 5,01 |
|  |  |  | 58 | -32 | 24 | 4,85 |
| Area 45 | L | 33 | -56 | 20 | -6 | 5,18 |
|  | 0 |  | -58 | 14 | -2 | 5,11 |
| IFG (p. Opercularis) | L |  | -58 | 12 | 2 | 5,02 |
| Superior Temporal Gyrus | R | 12 | 42 | -36 | 8 | 5,06 |
|  |  |  | 44 | -32 | 4 | 4,91 |
|  | 0 | 6 | 34 | 10 | 4 | 5,03 |
| Putamen | L | 5 | -32 | 0 | 2 | 5,03 |
| Cuneus | L | 8 | -12 | -88 | 38 | 4,98 |
| Postcentral Gyrus | R | 4 | 24 | -28 | 58 | 4,94 |
| Postcentral Gyrus | L | 5 | -58 | -14 | 20 | 4,93 |
| Precentral Gyrus | R | 1 | 52 | -4 | 50 | 4,91 |
| Posterior-Medial Frontal | R | 5 | 4 | -4 | 68 | 4,86 |
| Hippocampus | L | 2 | -26 | -26 | -10 | 4,84 |
| Rolandic Operculum | R | 3 | 62 | 6 | 8 | 4,83 |
| Postcentral Gyrus | L | 3 | -24 | -48 | 54 | 4,82 |
| Area 4p | R | 1 | 40 | -10 | 36 | 4,73 |
| Insula Lobe | R | 1 | 42 | 6 | 10 | 4,73 |
| Cerebelum (VI) | R | 1 | 14 | -62 | -18 | 4,73 |


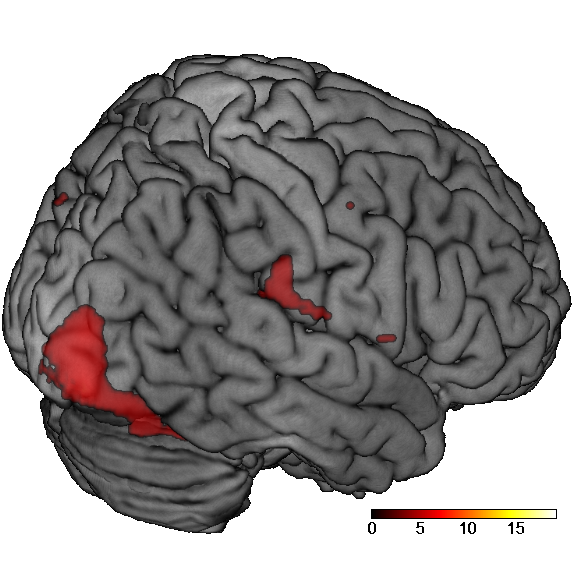

Supplement: Supplementary file 1 [file Table_1.DOCX]
